# Asymmetric Addition of Cyanide to β-Nitroalkenes Catalysed by Chiral Salen Complexes of Titanium(IV) and Vanadium(V)

**DOI:** 10.1002/cctc.201300215

**Published:** 2013-05-28

**Authors:** Michael North, James M Watson

**Affiliations:** [a] School of Chemistry, Newcastle UniversityBedson Building, Newcastle upon Tyne, NE1 7RU (UK), Fax: (+44) 1912226929

**Keywords:** asymmetric catalysis, Michael addition, N,O ligands, titanium, vanadium

## Abstract

Structurally well-defined bimetallic titanium(IV) (salen) and monometallic vanadium(V) (salen) complexes have been used as catalysts for the asymmetric addition of trimethylsilyl cyanide to β-nitroalkenes to produce chiral nitronitriles with *ee* values in the range of 79–89 % and conversions up to 100 % at 0 °C. The reaction conditions (solvent, temperature, time and vanadium complex counter-ion) were optimised, and it was shown that the catalyst loading could be significantly reduced (20 to 2 mol %) and the reaction temperature increased (−40 to 0 °C) compared to previous studies that used an in situ prepared catalyst. The results are compared and contrasted with previous results obtained by using the same catalysts for the asymmetric addition of trimethylsilyl cyanide to aldehydes, and a transition-state structure for the asymmetric addition of trimethylsilyl cyanide to nitroalkenes is proposed to account for the observed stereochemistry.

## Introduction

**Scheme 1:**
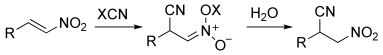
Conjugate addition of cyanide to nitroalkenes.

The asymmetric conjugate addition of cyanide to nitroalkenes to generate the corresponding non-racemic β-nitronitrile (Scheme 1) is a potentially useful route for the synthesis of a range of bi-functional compounds, such as β-amino acids,^1^ 1,3-diamines and 1,3-amino alcohols,^2^ which have potential bio-chemical and pharmaceutical applications.^3^ However, although a wide range of nucleophiles have been used in Michael additions to nitroalkenes,^4^–^12^ there are relatively few examples of cyanide addition to nitroalkenes reported in the literature. This is surprising given the synthetic versatility of both the nitro and nitrile functional groups and the apparently straightforward synthetic route; however, the reaction is complicated by the tendency of nitroalkenes to polymerise, by the ability of β-nitronitriles to eliminate either hydrogen cyanide^13^ or nitrous acid^14^ and by the possibility of the reaction occurring by either an anionic or radical-anion mechanism.^15^

**Figure 1:**
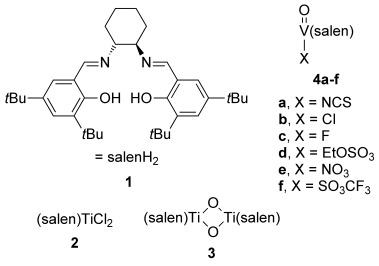
Salen ligands and complexes.

Synthetic conditions for the non-stereo-controlled addition of alkali metal cyanides to nitroalkenes were developed as long ago as 1947.^16^ In addition to alkali metal cyanides, acetone cyanohydrin^2^ or trimethylsilyl cyanide^17^ can also be used as the cyanide source. β-Nitronitriles can also be prepared by other methods that include the reaction of α-bromonitriles with nitronate anions^18^ and the conjugate addition of formaldehyde dimethylhydrazone to nitroalkenes followed by oxidation to the nitrile by using magnesium monoperoxyphthalate.^19^ The conjugate addition of cyanide to chiral nitroalkenes is known to occur diastereoselectively,^20^–^22^ however, there are only three reported examples of chiral catalysts for the enantioselective addition of cyanide to nitroalkenes.^1^, ^13^, ^23^ In 2008, Ricci and co-workers reported that quaternary ammonium salts obtained from cinchona alkaloids catalyse the asymmetric addition of cyanide from acetone cyanohydrin to β,β-di-substituted nitroalkenes under phase-transfer conditions to give β-nitronitriles with up to 72 % *ee*.^13^ Subsequently, Lassaletta and co-workers showed that a thiourea-substituted cinchona alkaloid quaternary ammonium salt with a cyanide counter-ion catalyses the asymmetric addition of trimethylsilyl cyanide to nitroalkenes with up to 86 % *ee*.^23^ The first metal-based catalyst for asymmetric β-nitronitrile synthesis was reported in 2012 by Wang and co-workers.^1^ It was shown that the in situ formed complex of salen ligand **1** (Figure 1) and titanium tetraisopropoxide catalyses the asymmetric addition of trimethylsilyl cyanide to nitroalkenes to give products with up to 84 % *ee*. However, it was necessary to use 20 mol % of the catalyst at −40 to −15 °C.

**Scheme 2:**
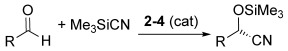
Asymmetric cyanohydrin synthesis.

We have previously reported that the same combination of **1** and titanium tetraisopropoxide catalyses the asymmetric addition of trimethylsilyl cyanide to aldehydes (Scheme 2).^24^, ^25^ It was also necessary to use 20 mol % of the catalyst at low temperatures to obtain good enantioselectivities in this reaction. Subsequently, we were able to prepare, isolate and structurally characterise the titanium(salen) dichloride complex **2**, which was a much more active catalyst for asymmetric cyanohydrin synthesis.^26^ Mechanistic studies^27^ showed that the active species in both of these systems was actually the bi-metallic complex **3** and just 0.1 mol % of **3** was able to catalyse the asymmetric addition of trimethylsilyl cyanide to aldehydes in less than one hour at room temperature.^28^, ^29^ Based on the mechanistic information obtained with titanium complexes,^27^, ^30^ we were able to develop vanadium(V)(salen) complexes **4** as even more enantioselective catalysts for the asymmetric addition of trimethylsilyl cyanide to aldehydes.^29^–^35^ In view of this precedent, we decided to investigate whether structurally well-defined metal(salen) complexes **3** and **4** might form highly active and enantioselective catalysts for the asymmetric addition of trimethylsilyl cyanide to nitroalkenes and in this paper we report the results of this work.

## Results and Discussion

**Scheme 3:**
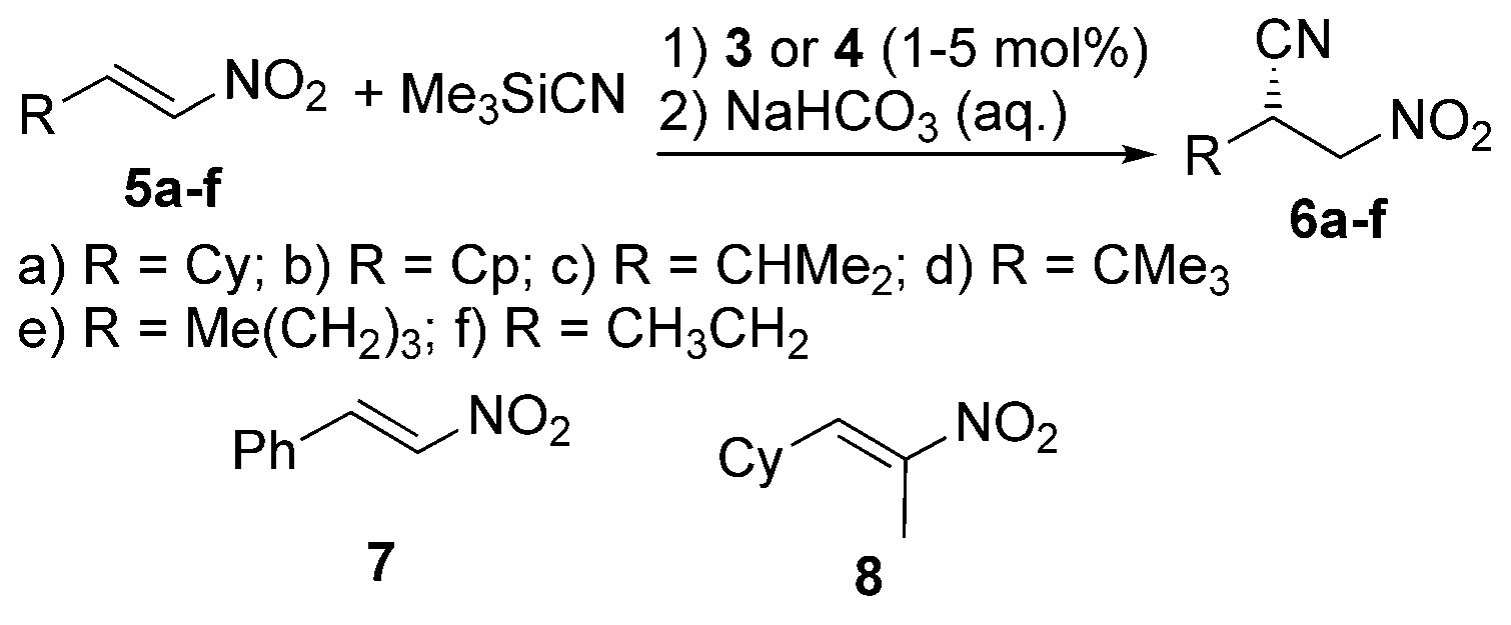
Asymmetric synthesis of 2-nitronitriles.

The reaction between nitroalkene **5 a** and trimethylsilyl cyanide (Scheme 3) was used for a screening study of the catalysts and reaction conditions, and the results are presented in Table 1. Titanium-based catalyst **3** and vanadium(V) catalyst **4 a** have been previously found to be the most active catalysts for asymmetric cyanohydrin synthesis,^28^–^35^ so they were used for this study. Catalyst **3** is an active catalyst for this reaction, and 100 % conversion of nitroalkene **5 a** into nitronitrile **6 a** with 62 % *ee* could be obtained at room temperature by using just 1 mol % of the catalyst with dichloromethane as the solvent (Table 1, entry 1). Under the same conditions, **4 a** was less reactive, but more enantioselective (entry 2); a trend that mirrors that seen in asymmetric cyanohydrin synthesis.^28^–^35^ The absolute configuration of **6 a** was determined as *S* if using catalyst **3** or **4 a** derived from (*R*,*R*)-salen ligand **1** by comparison of both the sign of its specific rotation and its chiral HPLC peak intensities with those reported in the literature.^1^ Thus, the structurally defined catalysts **3** and **4 a** give the same sense of asymmetric induction as the catalyst prepared in situ from **1** and titanium tetraisopropoxide.^1^ Lowering the reaction temperature to 0 °C resulted in an increase in the enantioselectivity if using **3** (entry 3), and increasing the amount of catalyst used to 2 mol % increased both the conversion and enantioselectivity obtained with both catalysts (entries 4 and 5). Further lowering the reaction temperature to −20 °C using catalyst **3** had a detrimental effect on the conversion, though the enantioselectivity increased further (entry 6). Increasing the amount of catalyst **3** to 5 mol % at room temperature or 0 °C significantly increased the rate of reaction but did not increase the enantioselectivity (entries 7 and 8).

**Table 1:** Synthesis of 6 a.

Entry	Catalyst	(Conc.)	*T*	*t*	Solvent	Conv.[a]	*ee*[b]
		[mol %]	[°C]	[h]		[%]	[%]
1	**3**	(1)	RT	18	CH_2_Cl_2_	100	62 (*S*)
2	**4 a**	(1)	RT	20	CH_2_Cl_2_	79	73 (*S*)
3	**3**	(1)	0	18	CH_2_Cl_2_	91	66 (*S*)
4	**3**	(2)	0	18	CH_2_Cl_2_	100	70 (*S*)
5	**4 a**	(2)	0	20	CH_2_Cl_2_	92	75 (*S*)
6	**3**	(2)	−20	18	CH_2_Cl_2_	30	84 (*S*)
7	**3**	(5)	RT	1	CH_2_Cl_2_	100	57 (*S*)
8	**3**	(5)	0	4	CH_2_Cl_2_	75	64 (*S*)
9	**3**	(2)	0	18	MePh	82	83 (*S*)
10	**3**	(2)	0	24	MePh	96	83 (*S*)
11	**3**	(3)	0	24	MePh	92	88 (*S*)
12	**3**	(5)	−20	72	MePh	0	–
13	**4 a**	(3)	0	24	MePh	83	83 (*S*)
14	**4 b**	(3)	0	24	MePh	76	80 (*S*)
15	**4 c**	(3)	0	24	MePh	77	86 (*S*)
16	**4 d**	(3)	0	24	MePh	40	n.d.
17	**4 e**	(3)	0	24	MePh	40	n.d.
18	**4 f**	(3)	0	24	MePh	41	n.d.

[a] Conversion determined by ^1^H NMR spectroscopy of the un-purified product.

[b] *ee* determined by chiral HPLC; n.d.=not determined. Absolute configuration determined by comparison of the specific rotation and HPLC peak intensities with literature data.^1^

Although dichloromethane had been previously found to be the optimal solvent for asymmetric cyanohydrin synthesis catalysed by **3** and **4 a**,^28^–^35^ Wang and co-workers showed that toluene was the optimal solvent for nitronitrile synthesis catalysed by the titanium complex of **1**.^1^ Therefore, the use of **3** and **4 a** in toluene was investigated. Reactions catalysed by **3** in toluene were slower than those in dichloromethane but more enantioselective (cf. Table 1, entries 4 and 9). By extending the reaction time to 24 h, the conversion increased to close to quantitative (entry 10), and increasing the amount of catalyst used to 3 mol % further increased the enantiomeric excess (*ee*) of the product to 88 % (entry 11). An attempt to lower the reaction temperature to −20 °C in toluene resulted in no reaction, even if 5 mol % of **3** was used with a reaction time of 72 h (entry 12).

In view of the lower intrinsic reactivity of **4 a**, its concentration was increased to 3 mol % in toluene, and at 0 °C this gave a good conversion to **6 a** with 83 % *ee* (entry 13). For asymmetric cyanohydrin synthesis catalysed by **4**, the structure of the anion significantly influences the catalytic activity, though not the enantioselectivity. The best results were obtained by using complexes with a nucleophilic counter-ion that was capable of acting as a Lewis base to activate the trimethylsilyl cyanide.^30^, ^32^, ^33^, ^35^ Therefore, **4 b**–**f** were screened as catalysts for the synthesis of **6 a** under the conditions of Table 1, entry 13. The results mirror those obtained for asymmetric cyanohydrin synthesis, with **4 a**–**c** giving higher conversions than **4 d**–**f** (entries 14–18), though there was little difference in the enantioselectivities observed with **4 a**–**c**.

The conditions of Table 1, entry 10 were taken as optimal for catalyst **3** (although the conditions of Table 1, entry 11 give the product with a slightly higher *ee*, this was at the expense of a 50 % increase in the amount of catalyst used), and the conditions of Table 1, entry 13 as optimal for catalyst **4 a**. These conditions were then used for the asymmetric synthesis of nitronitriles **6 b**–**f** (Table 2). The results were consistent both between the various nitroalkenes and between catalysts **3** and **4 a**. In all cases, nitronitriles were obtained with *ee* values of 80–89 %, and the reactions catalysed by **3** generally gave slightly higher conversions, so these reactions were worked up and the products purified to give isolated chemical yields of 77–93 %. Attempts to extend the chemistry to 2-nitrostyrene **7** were unsuccessful, consistent with the report of Wang and co-workers that only aliphatic nitroalkenes were substrates for the **1**/titanium tetraisopropoxide catalytic system.^1^ The 1,2-di-substituted nitroalkene (*E*)-2-cyclohexyl-1-methyl-nitroethene (**8**) also failed to react with trimethylsilyl cyanide in the presence of **3** or **4 a**. The absolute configurations of **6 a**,**c**–**f** were shown to be *S* by comparison of both specific rotation and chiral HPLC peak intensities with literature data.^1^, ^40^ Compound **6 b** has not been prepared before, but is assumed to have an *S* configuration based on the order of elution of its HPLC peaks and by analogy with **6 a**,**c**–**f**.

**Table 2:** Synthesis of 6 a–f.

Entry	Nitroalkene	Catalyst	Conv.[a]	Yield[b]	*ee*[c]
			[%]	[%]	[%]
1	**5 a**	**3**	96	84	83 (*S*)
2	**5 a**	**4 a**	83	–	83 (*S*)
3	**5 b**	**3**	96	77	88
4	**5 b**	**4 a**	80	–	84
5	**5 c**	**3**	94	90	86 (*S*)
6	**5 c**	**4 a**	88	–	83 (*S*)
7	**5 d**	**3**	100	85	80 (*S*)
8	**5 d**	**4 a**	75	–	79 (*S*)
9	**5 e**	**3**	93	81	89 (*S*)
10	**5 e**	**4 a**	74	–	89 (*S*)
11	**5 f**	**3**	96	93	85 (*S*)
12	**5 f**	**4 a**	87	–	79 (*S*)

[a] Conversion determined by ^1^H NMR spectroscopy of the un-purified product.

[b] Isolated yield of purified product.

[c] *ee* determined by chiral HPLC. Absolute configuration determined by comparison of the specific rotation and HPLC peak intensities with literature data.^1^, ^41^

**Figure 2:**
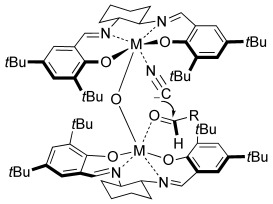
Transition state for M(salen)-catalysed asymmetric cyanohydrin synthesis. For reactions catalysed by 3, both M=Ti, whereas for reactions catalysed by 4, the M coordinated to the aldehyde is V^V^ and the M coordinated to the cyanide is V^IV^.

**Figure 3:**
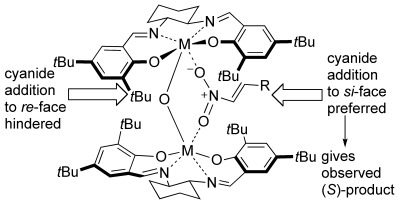
Possible transition state for M(salen)-catalysed asymmetric nitronitrile synthesis. M=Ti or V.

It is informative to compare the stereochemical outcomes of the use of **3** and **4 a** as catalysts for asymmetric cyanohydrin synthesis and nitronitrile synthesis. Extensive mechanistic work^27^–^36^ on the use of **3** and **4 a** as catalysts for asymmetric cyanohydrin synthesis has resulted in the transition-state model shown in Figure 2,^27^, ^33^ which shows that (for complexes derived from the (*R*,*R*)-salen ligand) cyanide addition occurs selectively on the *re* face of the coordinated aldehyde to lead to the (*S*)-cyanohydrin trimethylsilyl ether. In contrast, the results presented in this work show that the same complexes will catalyse the formation of (*S*)-nitronitriles, which requires cyanide addition to occur on the *si* face of the coordinated alkene. The opposite result would have been predicted had the nitroalkene simply coordinated to catalysts **3** and **4 a** in the same way as the aldehyde shown in Figure 2. More work is needed to fully elucidate the mechanism of this reaction and the origin of the asymmetric induction, but it is possible that the nitro group acts as a bidentate ligand to bridge the two metal ions. This would be analogous to the mechanism proposed for a cinchona–thiourea-based catalyst for the asymmetric addition of trimethylsilyl cyanide to nitroalkenes.^23^ A possible transition state is shown in Figure 3. In this structure, the orientation of the nitroalkene is determined by the stepped conformation^36^ of the salen ligands. This also results in the reaction of cyanide on the *si* face of the coordinated nitroalkene, which is less hindered than the reaction on the *re* face.

## Conclusions

By using pre-formed and structurally well-defined metal(salen) complexes, it is possible to significantly enhance the catalytic activity shown by metal(salen) complexes in the asymmetric addition of trimethylsilyl cyanide to aliphatic nitroalkenes. The literature procedure that used an in situ prepared catalyst obtained from **1** and titanium tetraisopropoxide required the use of 20 mol % of the catalyst at −40 rising to −15 °C. In contrast, just 2 mol % of catalyst **3** was able to achieve similar yields and enantioselectivities at 0 °C. Complexes **4 a**–**f** are the first vanadium-based catalysts to be reported for this reaction and the most active catalyst (**4 a**) was almost as active and just as enantioselective as titanium-based **3**, and 3 mol % of catalyst **4 a** at 0 °C gave the optimal conversions and enantioselectivities.

## Experimental Section

Catalysts **3** and **4 a**–**f** were prepared as previously reported.^32^, ^33^, ^37^ Nitroalkenes **5 a**–**f** were prepared by a *tert*-BuOK catalysed Henry reaction between the appropriate aldehyde and nitromethane followed by dehydration with Al_2_O_3_.^38^, ^39^ All other compounds were commercially available and used as supplied. ^1^H and ^13^C NMR spectra were recorded in CDCl_3_ at 25 °C by using a Bruker Avance300 spectrometer operating at 300 or 75 MHz, respectively, or a JEOL400 spectrometer operating at 400 or 100 MHz, respectively. Chemical shifts are quoted in ppm relative to tetramethylsilane. Mass spectra were measured by using a Waters LCT Premier LC–MS spectrometer. IR spectra were recorded by using a Varian 800 FTIR instrument. Melting points were determined by using a Stuart SMP3 system. Optical rotations were recorded in CHCl_3_ by using a Polaar 2001 Optical Activity automatic polarimeter and are reported as [*α*]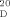 (concentration in g/100 mL). Enantiomeric excess values were determined by chiral HPLC by using ChiralPak OD or AD columns with a Varian Prostar HPLC apparatus with UV detection at 215 nm. Flash column chromatography was performed by using silica gel.

**General procedure for synthesis of racemic nitronitriles 6 a**–**f**: To a solution of nitroalkene **5 a**–**f** and achiral titanium(salen) complex^40^ analogous to **3** but derived from ethylenediamine (1 mol %) in CH_2_Cl_2_ (2 mL) was added Me_3_SiCN (1.2 equiv.). The reaction mixture was stirred at RT for 18 h, then the reaction was quenched with aqueous NaHCO_3_ and extracted with CH_2_Cl_2_ (3×10 mL). The combined organic layers were dried (MgSO_4_), and the solvent was removed under reduced pressure to leave an orange/yellow oil. This was dissolved in Et_2_O (5 mL) and filtered through silica eluting with Et_2_O (50 mL) to remove the catalyst. The eluent was evaporated under reduced pressure to give racemic samples of **6 a**–**f**.

**(*S*)-2-Cyclohexyl-3-nitropropanonitrile (6 a)**:^1^ To a solution of nitroalkene **5 a** (120 mg, 0.77 mmol) and catalyst **3** (18.8 mg 0.015 mmol) in toluene (2 mL) at 0 °C was added Me_3_SiCN (0.15 mL, 1.16 mmol). The reaction mixture was stirred at 0 °C for 24 h, then the reaction was quenched with aqueous NaHCO_3_ and extracted with CH_2_Cl_2_ (3×10 mL). The combined organic layers were dried (MgSO_4_), and the solvent was removed under reduced pressure to leave an orange/yellow oil, which was purified by flash column chromatography (petroleum ether/ethyl acetate 95:5) to give **6 a** (118 mg, 84 %) as a yellow oil with 83 % *ee* determined by chiral HPLC (using an OD column with hexane/*i*PrOH=95:5 at a flow rate of 1.0 mL min^−1^). *R*_t(major)_=31.8 min, *R*_t(minor)_=40.1 min; [*α*]
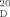
=−8.8 (*c*=0.53, CHCl_3_); lit.^1^ [*α*]
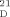
=−8.9 (*c*=1.01, CHCl_3_) for (*S*)-enantiomer; 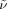
_max_(neat)=2929 (m), 2855 (m), 2246 (w), and 1557 cm^−1^ (s); ^1^H NMR (300 MHz, CDCl_3_): *δ*=1.1–1.4 (6 H, m), 1.6–1.9 (5 H, m), 3.34 (1 H, dt, ^3^*J*_HH_=8.8, 5.7 Hz), 4.55 (1 H, dd, ^2^*J*_HH_=13.9, ^3^*J*_HH_=6.2 Hz), 4.64 ppm (1 H, dd, ^2^*J*_HH_=13.9, ^3^*J*_HH_=8.1 Hz); ^13^C NMR (75 MHz, CDCl_3_): *δ*=25.5, 25.6, 25.8, 29.3. 31.1, 36.3, 37.8, 73.5, 117.0 ppm; MS (ESI): *m*/*z* (%): 205 [*M*+Na]^+^ (100), 196 (45), 194 (60), 147 (35), 102 (30).

**(*S*)-2-Cyclopentyl-3-nitropropanonitrile (6 b)**: To a solution of nitroalkene **5 b** (90 mg, 0.64 mmol) and catalyst **3** (15.5 mg, 0.013 mmol) in toluene (2 mL) at 0 °C was added Me_3_SiCN (0.15 mL, 1.16 mmol). The reaction mixture was stirred at 0 °C for 24 h, then the reaction was quenched with aqueous NaHCO_3_ and extracted with CH_2_Cl_2_ (3×10 mL). The combined organic layers were dried (MgSO_4_), and the solvent was removed under reduced pressure to leave an orange/yellow oil, which was purified by flash column chromatography (petroleum ether/ethyl acetate 95:5) to give **6 b** (83 mg, 77 %) as a yellow oil with 88 % *ee* determined by chiral HPLC (using an OD column with hexane/*i*PrOH=95:5 at a flow rate of 1.0 mL min^−1^). *R*_t(major)_=32.8 min, *R*_t(minor)_=38.5 min; [*α*]
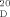
=−6.8 (*c*=1.0, CHCl_3_); 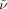
_max_(neat)=2961 (m), 2871 (m), 2247 (w), and 1557 cm^−1^ (s); ^1^H NMR (300 MHz, CDCl_3_): *δ*=1.3–1.5 (2 H, m), 1.6–1.7 (2 H, m), 1.7–1.8 (2 H, m), 1.8–2.0 (2 H, m), 2.0–2.2 (1 H, m), 3.44 (1 H, ddd, ^3^*J*_HH_=8.6, 6.6, 5.8 Hz), 4.52 (1 H, dd, ^2^*J*_HH_=13.8, ^3^*J*_HH_=5.8 Hz), 4.63 ppm (1 H, dd, ^2^*J*_HH_=13.8, ^3^*J*_HH_=8.5 Hz); ^13^C NMR (75 MHz, CDCl_3_): *δ*=25.0, 25.1, 29.8. 30.9, 35.1, 39.5, 74.7, 117.3 ppm; MS (ESI): *m*/*z* (%): 169 [*M*+H]^+^ (100), 143 (90), 130 (40), 122 (30), 114 (20), 102 (10); HRMS (ESI): *m*/*z* calcd for C_8_H_13_N_2_O_2_+H^+^: 169.0977; found: 169.0983.

**(*S*)-3-Methyl-2-(nitromethyl)butanonitrile (6 c)**:^1^ To a solution of nitroalkene **5 c** (90 mg, 0.78 mmol) and catalyst **3** (19.0 mg, 0.016 mmol) in toluene (2 mL) at 0 °C was added Me_3_SiCN (0.12 mL, 0.94 mmol). The reaction mixture was stirred at 0 °C for 24 h, then the reaction was quenched with aqueous NaHCO_3_ and extracted with CH_2_Cl_2_ (3×10 mL). The combined organic layers were dried (MgSO_4_), and the solvent was removed under reduced pressure to leave an orange/yellow oil, which was purified by flash column chromatography (petroleum ether/ethyl acetate 95:5) to give **6 c** (100 mg, 90 %) as a yellow oil with 86 % *ee* determined by chiral HPLC (using an OD column with hexane/*i*PrOH=95:5 at a flow rate of 1.0 mL min^−1^). *R*_t(major)_=33.2 min, *R*_t(minor)_=40.9 min; [*α*]
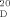
=+4.0 (*c*=0.65, CHCl_3_); lit.^1^ [*α*]
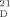
=+3.8 (*c*=1.05, CHCl_3_) for (*S*)-enantiomer; 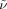
_max_(neat): 2970 (m), 2245 (w), and 1557 cm^−1^ (s); ^1^H NMR (300 MHz, CDCl_3_): *δ*=1.14 (3 H, d, ^3^*J*_HH_=6.7 Hz), 1.17 (3 H, d, ^3^*J*_HH_=6.8 Hz), 2.02 (1 H, sept d, ^3^*J*_HH_=7.0, 5.0 Hz), 3.38 (1 H, ddd, ^3^*J*_HH_=8.3, 6.4, 5.0 Hz), 4.53 (1 H, dd, ^2^*J*_HH_=13.9, ^3^*J*_HH_=6.2 Hz), 4.65 ppm (1 H, dd, ^2^*J*_HH_=13.9, ^3^*J*_HH_=8.2 Hz); ^13^C NMR (100 MHz, CDCl_3_): *δ*=18.3, 20.7, 28.5, 36.9, 73.6, 116.8 ppm; MS (ESI): *m*/*z* (%): 165 [*M*+Na]^+^ (100), 151 (95), 147 (95), 102 (70).

**(*S*)-3,3-Dimethyl-2-(nitromethyl)butanenitrile (6 d)**:^41^ To a solution of nitroalkene **5 d** (51 mg, 0.39 mmol) and catalyst **3** (12.8 mg, 0.011 mmol) in toluene (2 mL) at 0 °C was added Me_3_SiCN (0.08 mL, 0.63 mmol). The reaction mixture was stirred at 0 °C for 24 h, then the reaction was quenched with aqueous NaHCO_3_ and extracted with CH_2_Cl_2_ (3×10 mL). The combined organic layers were dried (MgSO_4_), and the solvent was removed under reduced pressure to leave an orange/yellow oil, which was purified by flash column chromatography (petroleum ether/ethyl acetate 95:5) to give **6 d** (52 mg, 85 %) as a yellow solid with 80 % *ee* determined by chiral HPLC (using an AD column with hexane/*i*PrOH=99:1 at a flow rate of 1.5 mL min^−1^). *R*_t(minor)_=15.3 min, *R*_t(major)_=16.3 min; m.p. 112–114 °C; [*α*]
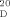
=+32.0 (*c*=0.1, CHCl_3_); lit.^41^ [*α*]
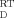
=+44.7 (*c*=1.01, CHCl_3_) for (*S*)-enantiomer; 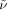
_max_(neat)=2961 (m), 2874 (w), 2243 (w), and 1558 cm^−1^ (s); ^1^H NMR (300 MHz, CDCl_3_): *δ*=1.16 (9 H, s), 3.28 (1 H, t, ^3^*J*_HH_=7.6 Hz), 4.58 ppm (2 H, d, ^3^*J*_HH_=7.6 Hz); ^13^C NMR (75 MHz, CDCl_3_): *δ*=27.3, 33.4, 41.7, 73.1, 117.5 ppm; MS (ESI): *m*/*z* (%): 179 [*M*+Na]^+^ (100), 173 (35), 167 (30), 151 (40), 102 (50).

**(*S*)-2-(Nitromethyl)hexanonitrile (6 e)**:^1^ To a solution of nitroalkene **5 e** (68 mg, 0.53 mmol) and catalyst **3** (9.6 mg, 0.0079 mmol) in toluene (2 mL) at 0 °C was added Me_3_SiCN (0.06 mL, 0.47 mmol). The reaction mixture was stirred at 0 °C for 24 h, then the reaction was quenched with aqueous NaHCO_3_ and extracted with CH_2_Cl_2_ (3×10 mL). The combined organic layers were dried (MgSO_4_), and the solvent was removed under reduced pressure to leave an orange/yellow oil, which was purified by flash column chromatography (petroleum ether/ethyl acetate 95:5) to give **6 e** (67 mg, 81 %) as a yellow oil with 89 % *ee* determined by chiral HPLC (using an OD column with hexane/*i*PrOH=95:5 at a flow rate of 1.0 mL min^−1^). *R*_t(major)_=31.2 min, *R*_t(minor)_=35.6 min; [*α*]
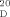
=−18.4 (*c*=0.97, CHCl_3_); lit.^1^ [*α*]
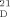
=−19.8 (*c*=1.01, CHCl_3_) for (*S*)-enantiomer; 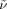
_max_(neat)=2961 (m), 2873 (w), 2248 (w), and 1557 cm^−1^ (s); ^1^H NMR (300 MHz, CDCl_3_): *δ*=0.94 (3 H, t, ^3^*J*_HH_=7.2 Hz), 1.3–1.8 (6 H, m), 3.40 (1 H, tt, ^3^*J*_HH_=8.1, ^3^*J*_HH_=5.9 Hz), 4.51 (1 H, dd, ^2^*J*_HH_=14.0, ^3^*J*_HH_=6.2 Hz), 4.63 ppm (1 H, dd, ^2^*J*_HH_=14.0, ^3^*J*_HH_=8.8 Hz); ^13^C NMR (75 MHz, CDCl_3_): *δ*=13.4, 21.9, 28.7, 29.3, 29.9, 74.8, 117.8; MS (ESI): *m*/*z* (%): 179 [*M*+Na]^+^ (100), 159 (30), 150 (40), 147 (60), 102 (65).

**(*S*)-2-(Nitromethyl)butanonitrile (6 f)**:^41^ To a solution of nitroalkene **5 f** (58 mg, 0.57 mmol) and catalyst **3** (13.9 mg, 0.011 mmol) in toluene (2 mL) at 0 °C was added Me_3_SiCN (0.1 mL, 0.85 mmol). The reaction mixture was stirred at 0 °C for 24 h, then the reaction was quenched with aqueous NaHCO_3_ and extracted with CH_2_Cl_2_ (3×10 mL). The combined organic layers were dried (MgSO_4_) and the solvent was removed under reduced pressure to leave an orange/yellow oil, which was purified by flash column chromatography (petroleum ether/ethyl acetate 95:5) to give **6 f** (68 mg, 93 %) as a yellow oil with 85 % *ee* determined by chiral HPLC (using an OD column with hexane/*i*PrOH=98:2 at a flow rate of 1.5 mL min^−1^). *R*_t(major)_=43.5 min, *R*_t(minor)_=49.9 min; [*α*]
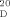
=−10.6 (*c*=0.5, CHCl_3_); lit.^41^ [*α*]
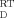
=−101.2 (*c*=0.98, CHCl_3_) for (*S*)-enantiomer; 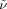
_max_(neat)=2961 (m), 2873 (w), 2248 (w), and 1557 cm^−1^ (s); ^1^H NMR (400 MHz, CDCl_3_): *δ*=1.20 (3 H, t, ^3^*J*_HH_=7.4 Hz), 1.7–1.9 (2 H, m), 3.39 (1 H, tt, ^3^*J*_HH_=8.0, 6.1 Hz), 4.52 (1 H, dd, ^2^*J*_HH_=13.9, ^3^*J*_HH_=6.4 Hz), 4.66 ppm (1 H, dd, ^2^*J*_HH_=13.9, ^3^*J*_HH_=7.6 Hz); ^13^C NMR (100 MHz, CDCl_3_): *δ*=11.1, 23.0, 31.3, 74.3, 117.7 ppm; MS (ESI): *m*/*z* (%): 151 [*M*+Na]^+^ (100), 135 (40), 102 (20).

## Notes

As a service to our authors and readers, this journal provides supporting information supplied by the authors. Such materials are peer reviewed and may be re-organized for online delivery, but are not copy-edited or typeset. Technical support issues arising from supporting information (other than missing files) should be addressed to the authors.

## References

[1] Lin L, Yin W, Fu X, Zhang J, Ma X, Wang R (2012). Org. Biomol. Chem.

[2] Anderson JC, Blake AJ, Mills M, Ratcliffe PD (2008). Org. Lett.

[3] Qiu JX, Petersson EJ, Matthews EE, Schepartz A (2006). J. Am. Chem. Soc.

[4] Berner OM, Tedeschi L, Enders D (2002). Eur. J. Org. Chem.

[5] Duursma A, Minnaard AJ, Feringa BL (2003). J. Am. Chem. Soc.

[6] Ballini R, Bosica G, Fiorini D, Palmieri A, Petrini M (2005). Chem. Rev.

[7] Enders D, Bonten MH, Raabe G (2007). Angew. Chem.

[8] Wu L-Y, Yan Z-Y, Xie Y-X, Niu Y-N, Liang Y-M (2007). Tetrahedron: Asymmetry.

[9] Wang C, Yu C, Liu C, Peng Y (2009). Tetrahedron Lett.

[10] Boyce GR, Johnson JS (2010). Angew. Chem.

[11] Kilic H, Bayindir S, Erdogan E, Saracoglu N (2012). Tetrahedron.

[12] Wu Y, Wang J, Li P, Kwong FY (2012). Synlett.

[13] Bernardi L, Fini F, Fochi M, Ricci A (2008). Synlett.

[14] Hong S-J, Lee C-H (2012). Tetrahedron Lett.

[15] Gross Z, Hoz S (1988). J. Am. Chem. Soc.

[16] Buckley GD, Heath RL, Rose JD (1947). J. Chem. Soc.

[17] Yadav LDS, Rai A (2009). Tetrahedron Lett.

[18] Ros F, de La Rosa J (1988). J. Org. Chem.

[19] Lassaletta J-M, Fernández R, Gasch C, Vázquez J (1996). Tetrahedron.

[20] McKenna J, McKenna JM, Smith PB (1965). Tetrahedron.

[21] Sakakibara T, Tachimori Y, Sudoh R (1982). Tetrahedron Lett.

[22] Sakakibara T, Tachimori Y, Sudoh R (1984). Tetrahedron.

[23] Bernal P, Fernández R, Lassaletta JM (2010). Chem. Eur. J.

[24] Belokon′ Y, Flego M, Ikonnikov N, Moscalenko M, North M, Orizu C, Tararov V, Tasinazzo M (1997). J. Chem. Soc. Perkin Trans. 1.

[25] Tararov VI, Orizu C, Ikonnikov NS, Larichev VS, Moscalenko MA, Yashkina LV, North M, Belokon′ YN (1999). Russ. Chem. Bull.

[26] Tararov VI, Hibbs DE, Hursthouse MB, Ikonnikov NS, Malik KMA, North M, Orizu C, Belokon′ YN (1998). Chem. Commun.

[27] Belokon′ YN, Green B, Ikonnikov NS, Larichev VS, Lokshin BV, Moscalenko MA, North M, Orizu C, Peregudov AS, Timofeeva GI (2000). Eur. J. Org. Chem.

[28] Belokon′ YN, Caveda-Cepas S, Green B, Ikonnikov NS, Khrustalev VN, Larichev VS, Moscalenko MA, North M, Orizu C, Tararov VI, Tasinazzo M, Timofeeva GI, Yashkina LV (1999). J. Am. Chem. Soc.

[29] Belokon′ YN, Green B, Ikonnikov NS, North M, Parsons T, Tararov VI (2001). Tetrahedron.

[30] North M, Omedes-Pujol M, Williamson C (2010). Chem. Eur. J.

[31] Belokon′ YN, North M, Parsons T (2000). Org. Lett.

[32] Belokon′ YN, Maleev VI, North M, Usanov DL (2006). Chem. Commun.

[33] Belokon′ YN, Clegg W, Harrington RW, Maleev VI, North M, Pujol MO, Usanov DL, Young C (2009). Chem. Eur. J.

[34] Chechik V, Conte M, Dransfield T, North M, Omedes-Pujol M (2010). Chem. Commun.

[35] North M, Omedes-Pujol M (2010). Beilstein J. Org. Chem.

[36] Katsuki T (2002). Adv. Synth. Catal.

[37] Belokon′ YN, Carta P, Gutnov AV, Maleev V, Moskalenko MA, Yashkina LV, Ikonnikov NS, Voskoboev NV, Khrustalev VN, North M (2002). Helv. Chim. Acta.

[38] Ballini R, Castagnani R, Petrini M (1992). J. Org. Chem.

[39] DiRocco DA, Oberg KM, Dalton DM, Rovis T (2009). J. Am. Chem. Soc.

[40] Belokon′ YN, Clegg W, Harrington RW, Young C, North M (2007). Tetrahedron.

[41] Enders D, Syrig R, Raabe G, Fernández R, Gasch C, Lassaletta J-M, Llera J-M (1996). Synthesis.

