# Supplementary material for: Asymmetric Addition of Cyanide to β-Nitroalkenes Catalysed by Chiral Salen Complexes of Titanium(IV) and Vanadium(V)
Source: ChemCatChem. 2013 May 28;5(8):2405–9. doi: 10.1002/cctc.201300215 (PMC3798126; doi:10.1002/cctc.201300215)
Supplement: Supplementary file 1 [file cctc0005-2405-sd1.pdf]

Heterogeneous & Homogeneous & Bio-
CHEMCATCHEM
CATALYSIS

Supporting Information

© Copyright Wiley-VCH Verlag GmbH & Co. KGaA, 69451 Weinheim, 2013

Asymmetric Addition of Cyanide to β-Nitroalkenes Catalysed by Chiral Salen Complexes of Titanium(IV) and Vanadium(V)

Michael North* and James M. Watson[a]

cctc_201300215_sm_miscellaneous_information.pdf

Contents

(*S*)–2–Cyclohexyl–3–nitropropanonitrile (6a)
^{1}H NMR spectrum 2
^{13}C NMR spectrum 3
Chiral HPLC traces 4

(*S*)–2–Cyclopentyl–3–nitropropanonitrile (6b)
^{1}H NMR spectrum 5
^{13}C NMR spectrum 6
Chiral HPLC traces 7

(*S*)-3-Methyl-2-(nitromethyl)butanonitrile (6c)
^{1}H NMR spectrum 8
^{13}C NMR spectrum 9
Chiral HPLC traces 10

(*S*)-3,3-Dimethyl-2-(nitromethyl)butanenitrile (6d)
^{1}H NMR spectrum 11
^{13}C NMR spectrum 12
Chiral HPLC traces 13

(*S*)-2-(Nitromethyl)hexanonitrile (6e)
^{1}H NMR spectrum 14
^{13}C NMR spectrum 15
Chiral HPLC traces 16

(*S*)-2-(Nitromethyl)butanonitrile (6f)
^{1}H NMR spectrum 17
^{13}C NMR spectrum 18
Chiral HPLC traces 19

(*S*)–2–Cyclohexyl–3–nitropropanonitrile (6a) ^{1}H NMR spectrum

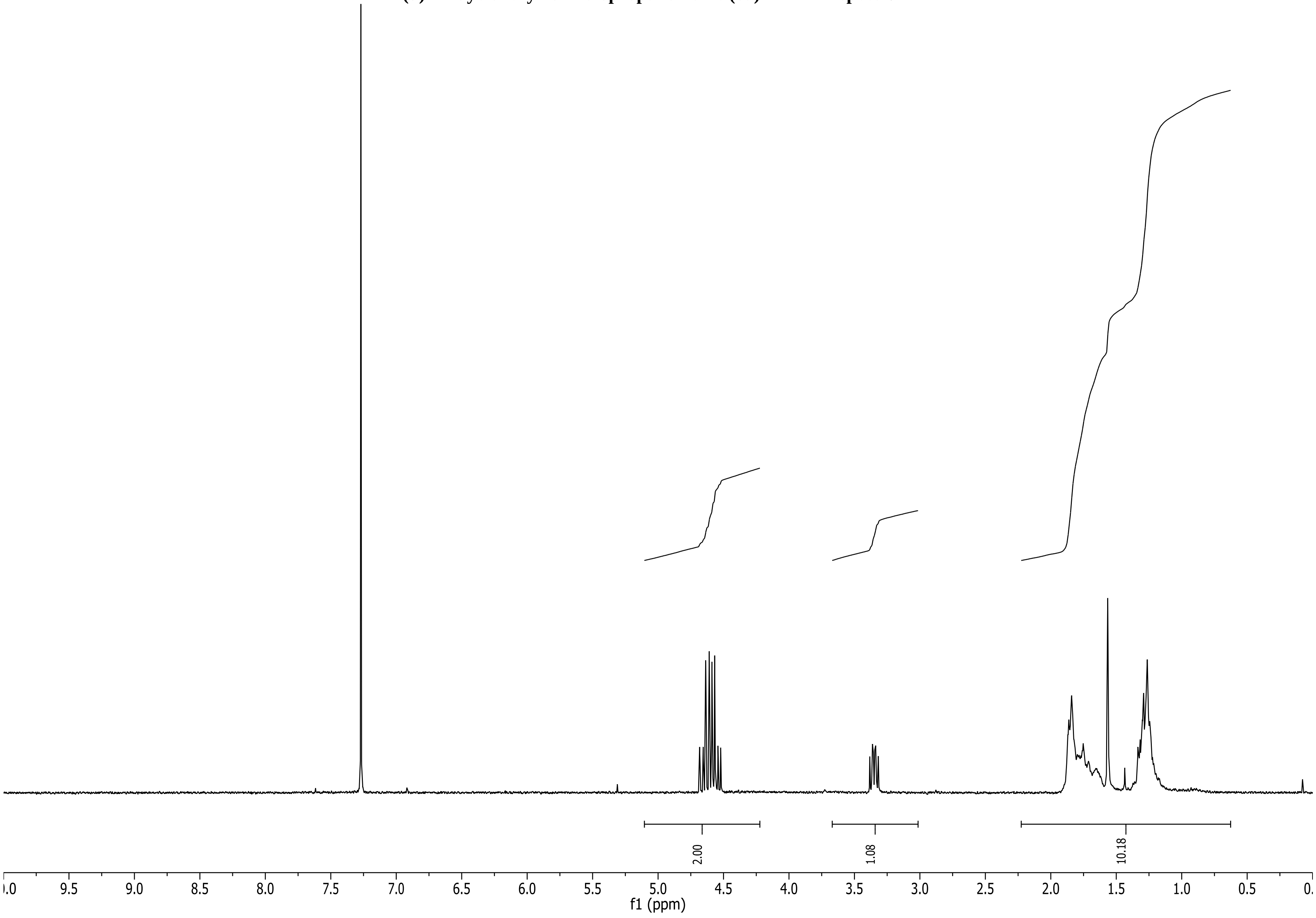

(*S*)–2–Cyclohexyl–3–nitropropanonitrile (6a) ^{13}C NMR spectrum

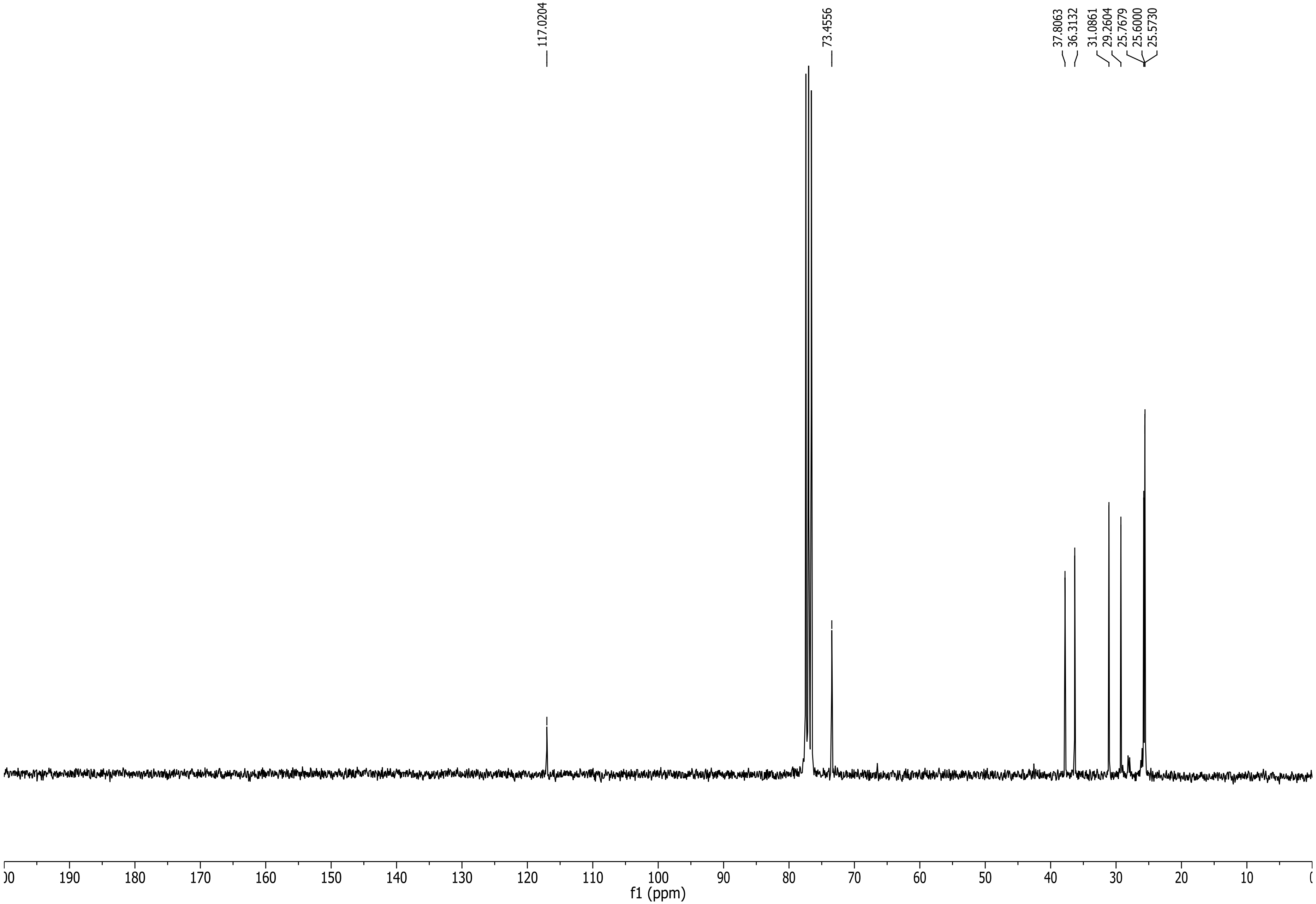

(*S*)–2–Cyclohexyl–3–nitropropanonitrile (6a) Chiral HPLC traces

Racemic

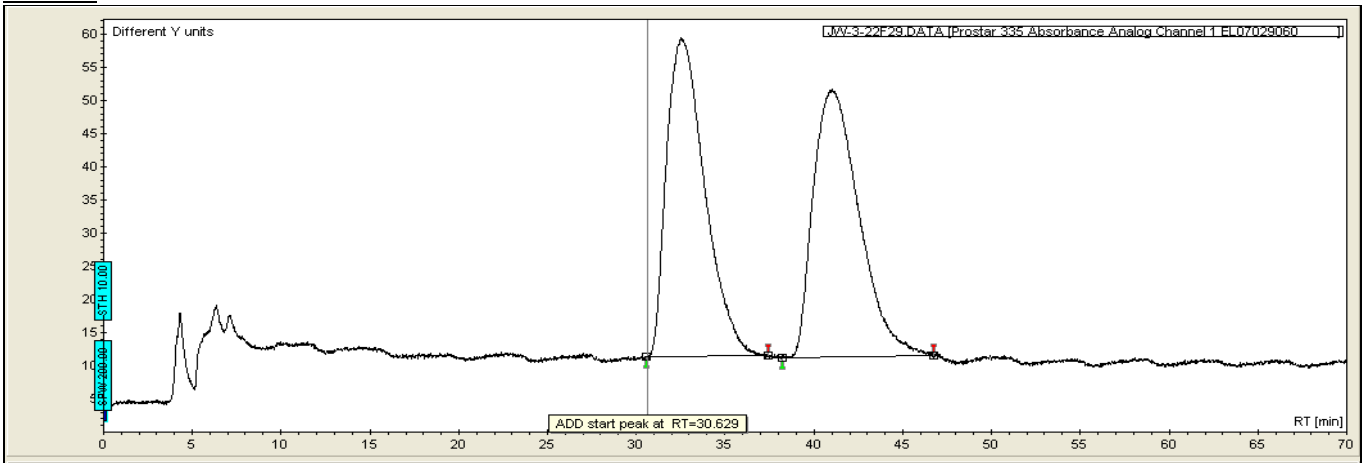

#	Name	Time [Min]	Quantity [% Area]	Height [mAU]	Area [mAU.Min]	Area % [%]
1	UNKNOWN	32.51	49.91	48.1	120.1	49.910
2	UNKNOWN	41.01	50.09	40.4	120.5	50.090
Total			100.00	88.5	240.6	100.000

Chiral prepared using catalyst **3**

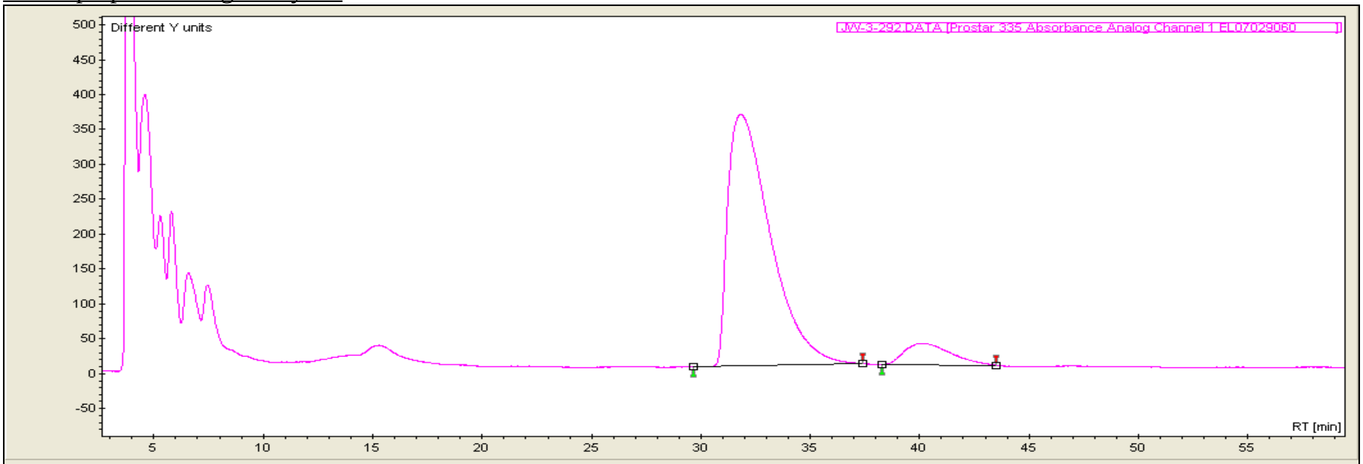

#	Name	Time [Min]	Quantity [% Area]	Height [mAU]	Area [mAU.Min]	Area % [%]
1	UNKNOWN	31.80	91.54	361.0	812.4	91.543
2	UNKNOWN	40.09	8.46	30.6	75.1	8.457
Total			100.00	391.6	887.5	100.000

Chiral prepared using catalyst **4a**

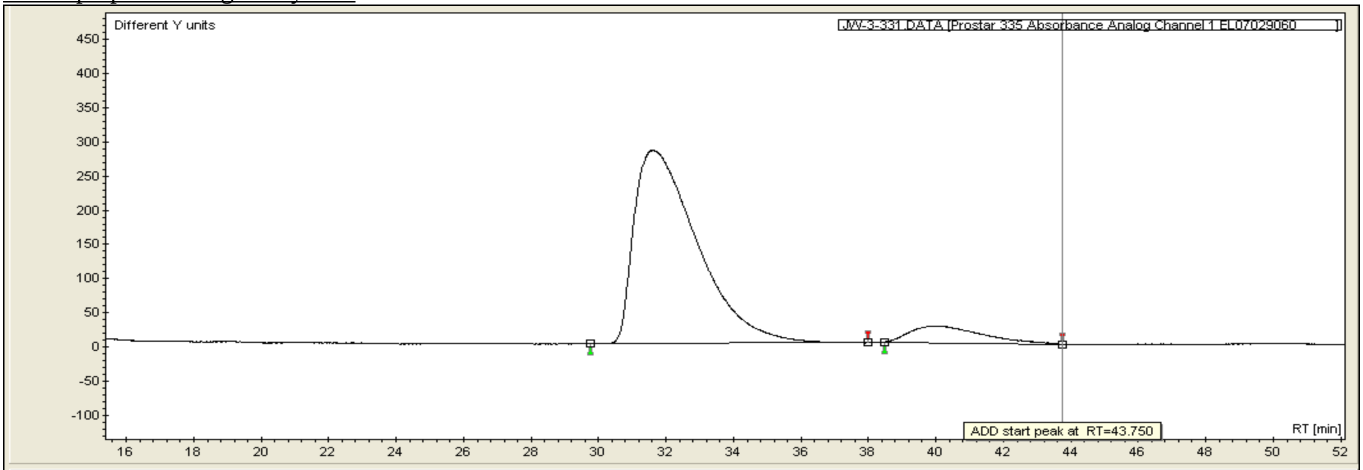

#	Name	Time [Min]	Quantity [% Area]	Height [mAU]	Area [mAU.Min]	Area % [%]
1	UNKNOWN	31.61	91.31	283.0	614.3	91.314
2	UNKNOWN	39.99	8.69	24.0	58.4	8.686
Total			100.00	307.0	672.7	100.000

(*S*)–2–Cyclopentyl–3–nitropropanonitrile (6b) ^{1}H NMR spectrum

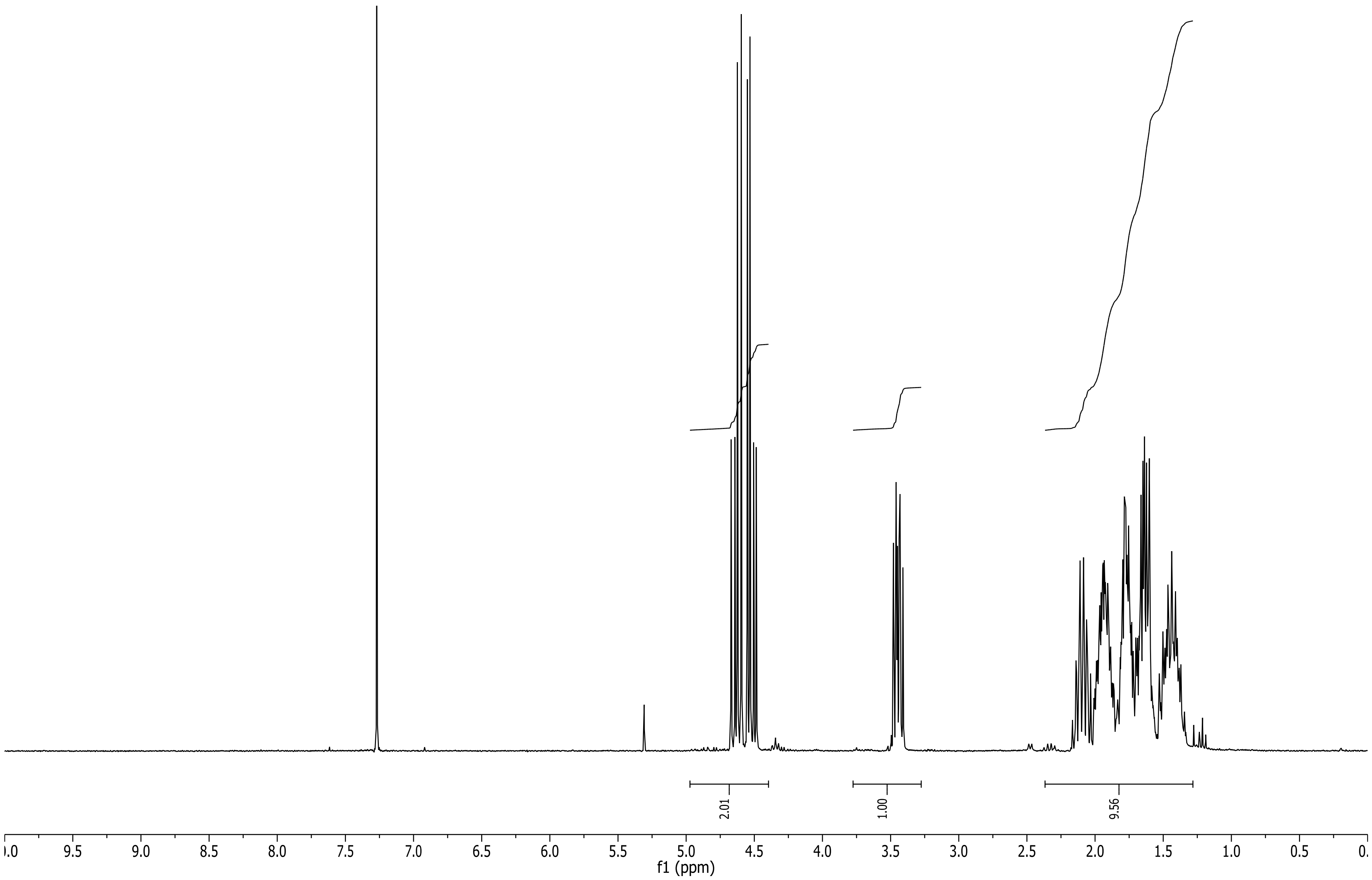

(*S*)–2–Cyclopentyl–3–nitropropanonitrile (6b) ^{13}C NMR spectrum

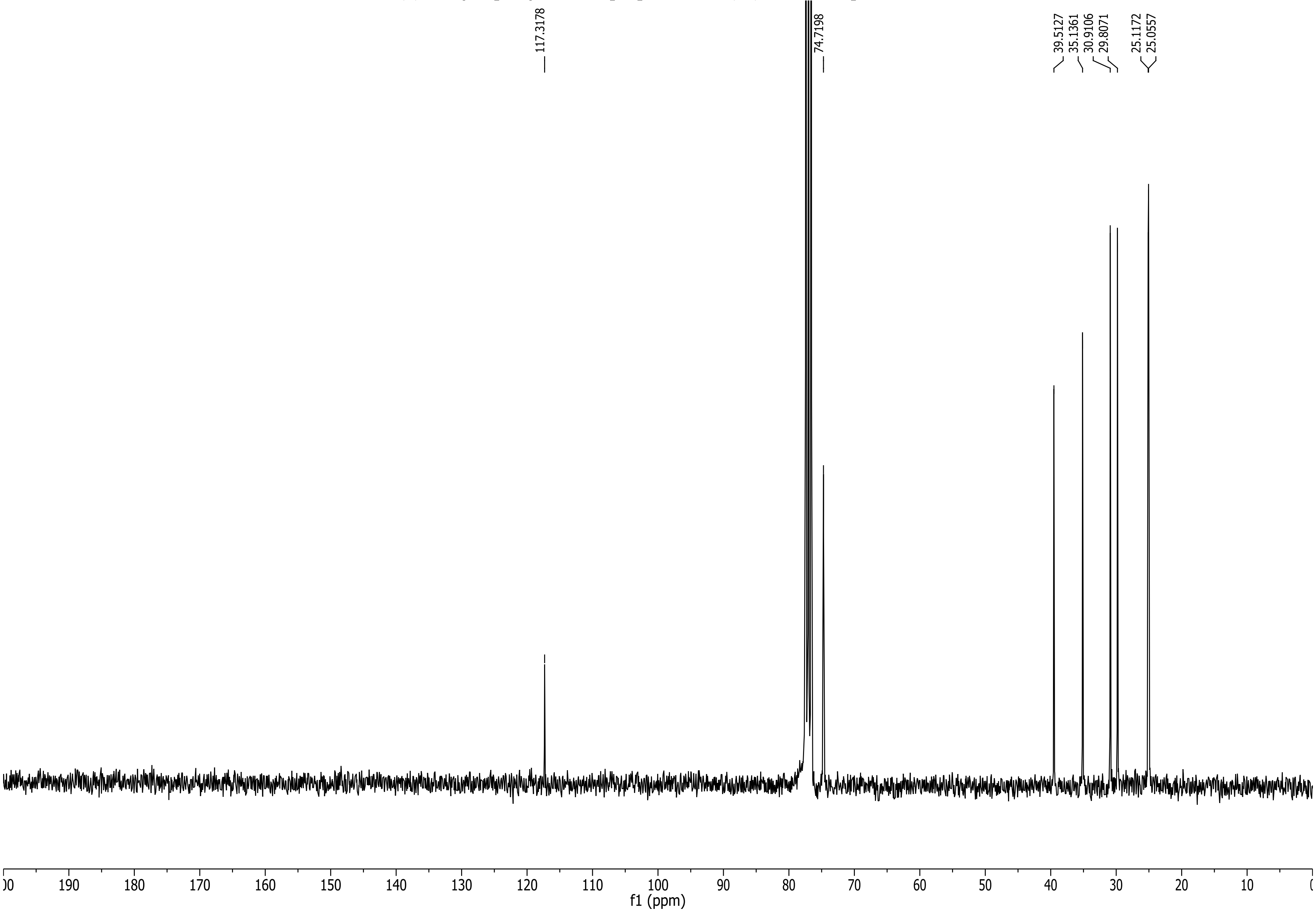

(*S*)–2–Cyclopentyl–3–nitropropanonitrile (6b) Chiral HPLC traces

Racemic

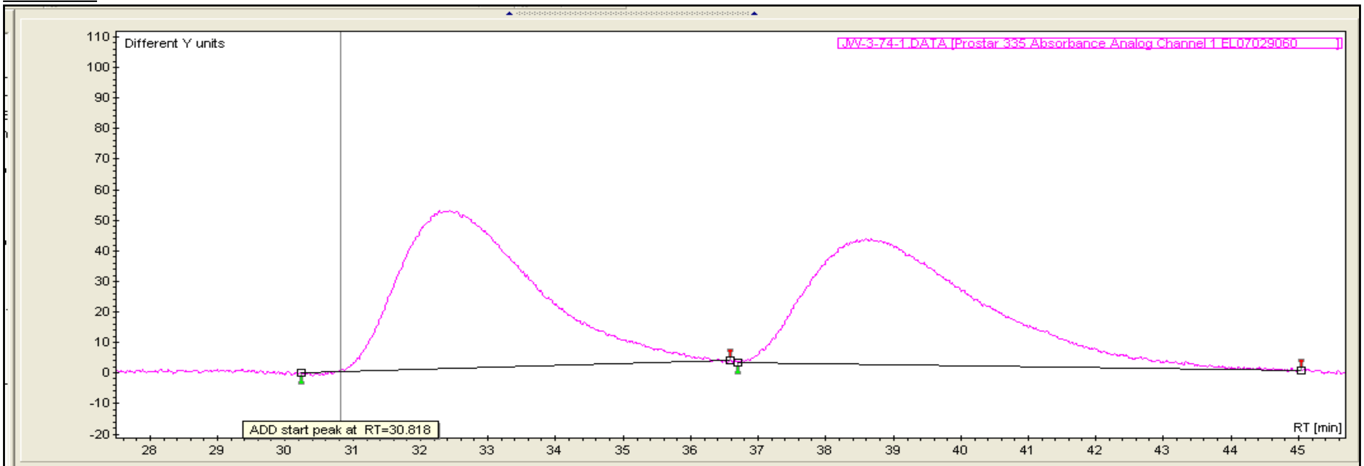

#	Name	Time [Min]	Quantity [% Area]	Height [mAU]	Area [mAU.Min]	Area % [%]
1	UNKNOWN	32.41	49.77	51.4	120.3	49.766
2	UNKNOWN	38.68	50.23	40.9	121.4	50.234
Total			100.00	92.4	241.8	100.000

Chiral prepared using catalyst **3**

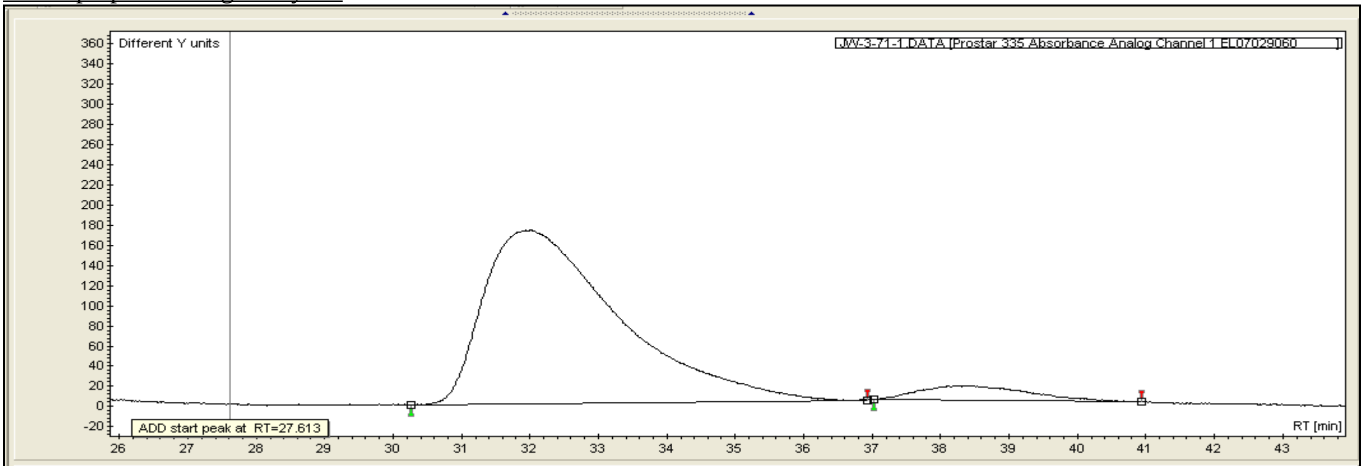

#	Name	Time [Min]	Quantity [% Area]	Height [mAU]	Area [mAU.Min]	Area % [%]
1	UNKNOWN	32.01	93.83	171.9	392.7	93.827
2	UNKNOWN	38.45	6.17	13.8	25.8	6.173
Total			100.00	185.8	418.6	100.000

Chiral prepared using catalyst **4a**

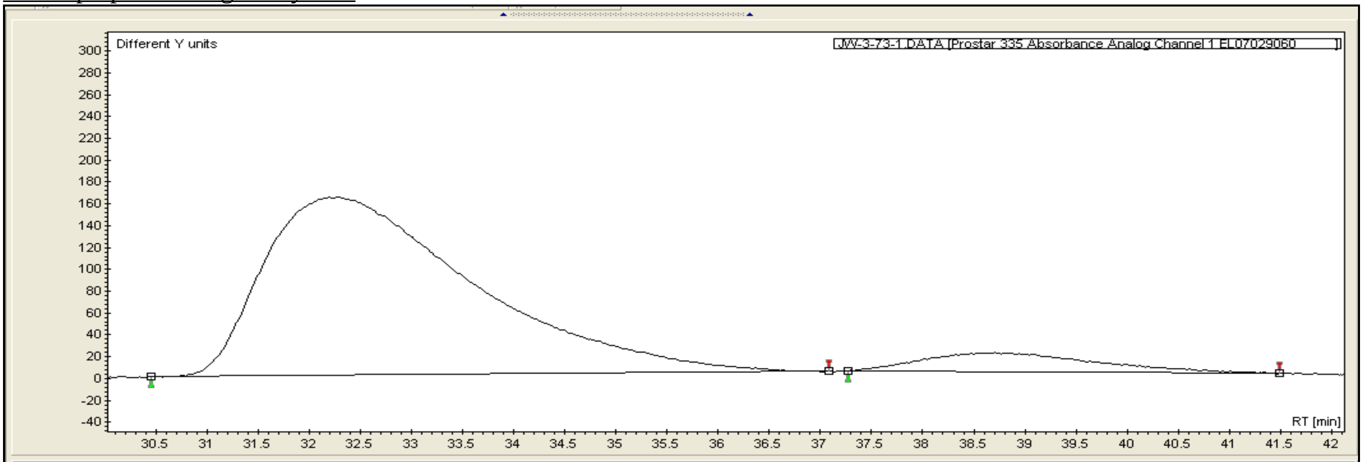

#	Name	Time [Min]	Quantity [% Area]	Height [mAU]	Area [mAU.Min]	Area % [%]
1	UNKNOWN	32.20	91.95	163.5	389.2	91.948
2	UNKNOWN	38.71	8.05	17.4	34.1	8.052
Total			100.00	180.9	423.3	100.000

(*S*)-3-Methyl-2-(nitromethyl)butanonitrile (6c) ^{1}H NMR spectrum

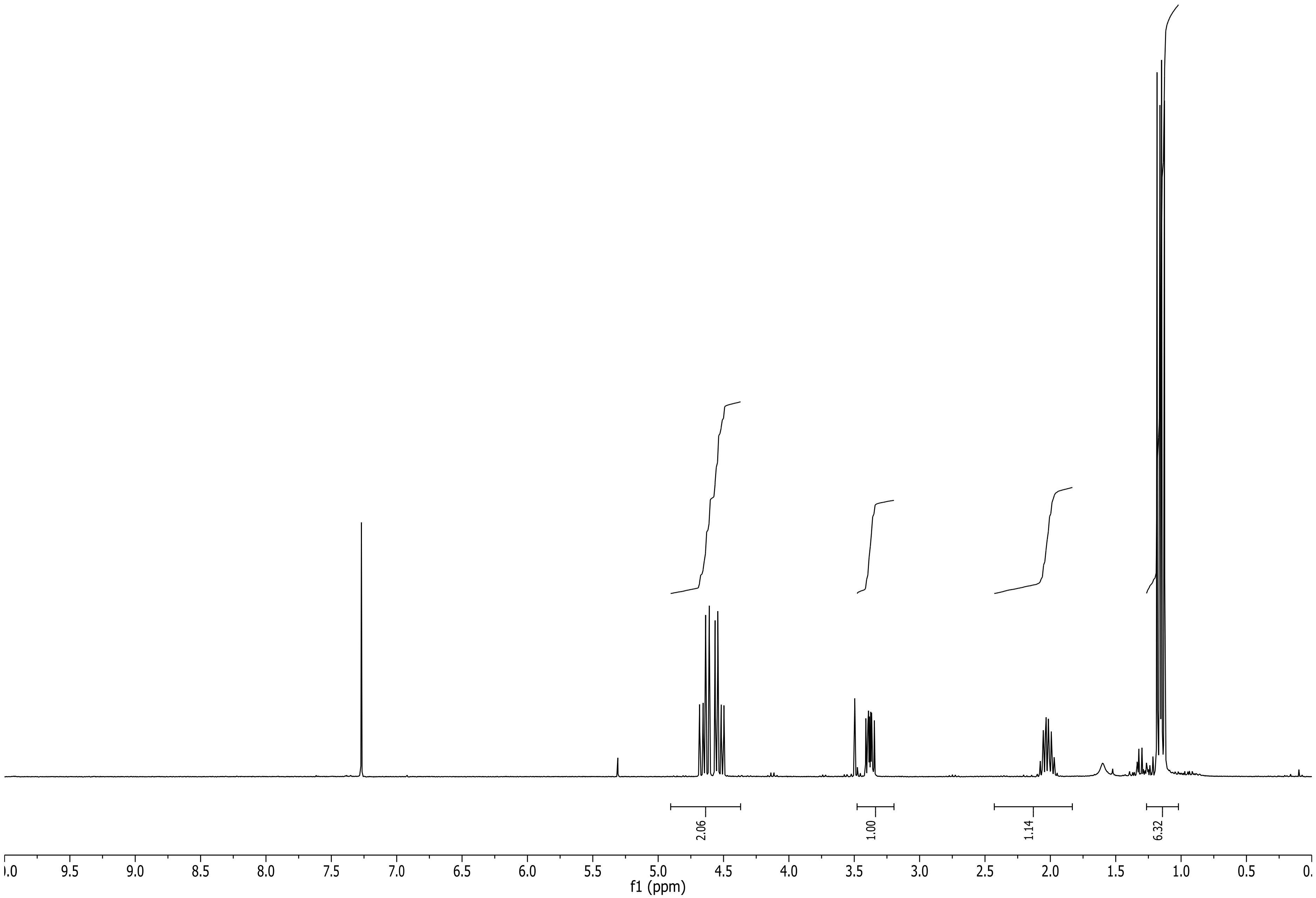

(*S*)-3-Methyl-2-(nitromethyl)butanonitrile (6c) ^{13}C NMR spectrum

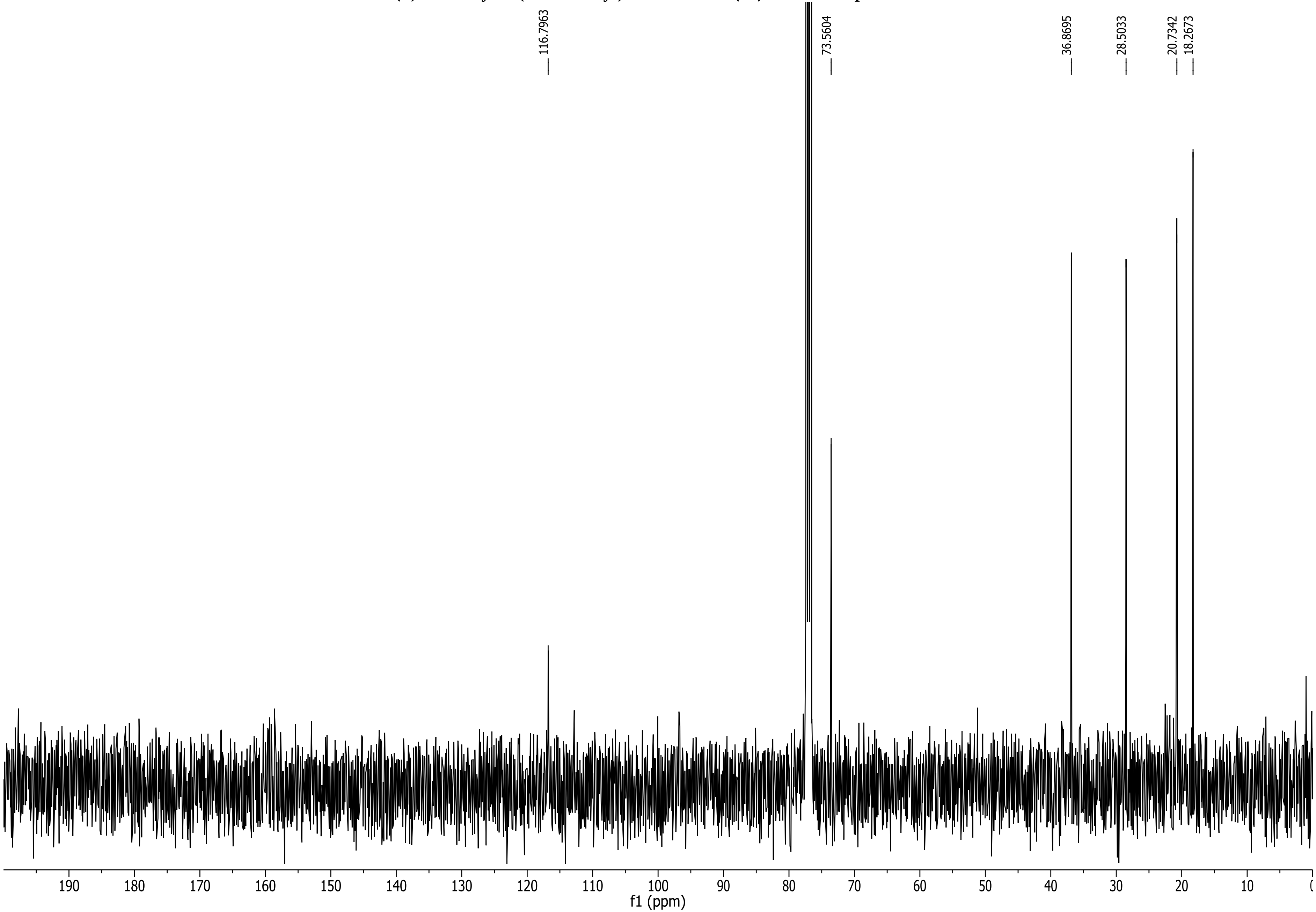

(*S*)-3-Methyl-2-(nitromethyl)butanonitrile (6c) Chiral HPLC traces

Racemic

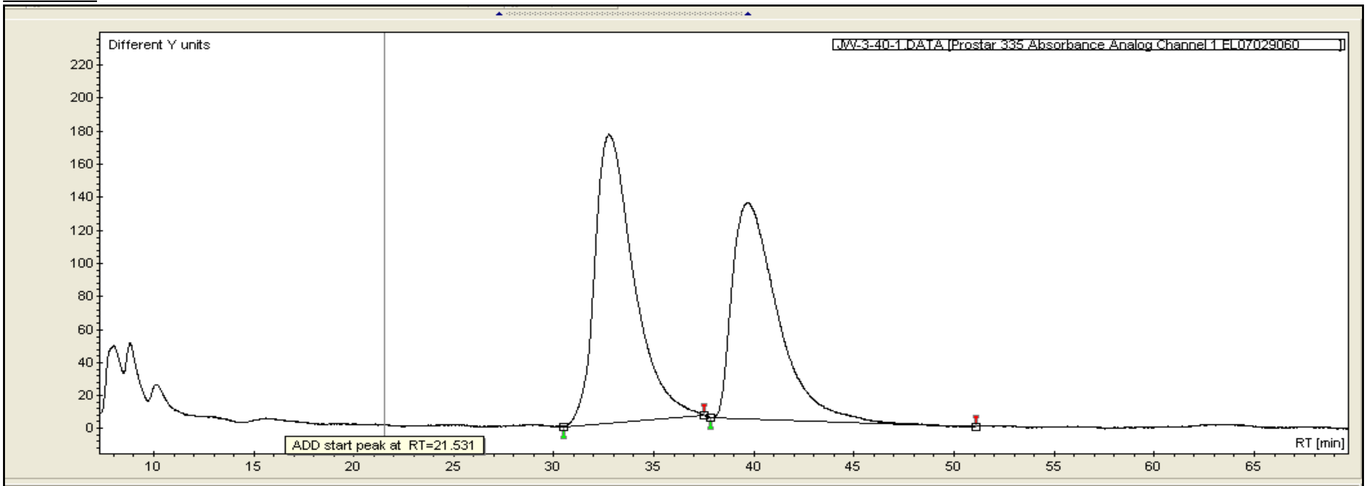

#	Name	Time [Min]	Quantity [% Area]	Height [mAU]	Area [mAU.Min]	Area % [%]
2	UNKNOWN	32.76	53.64	174.4	394.4	53.636
1	UNKNOWN	39.68	46.36	130.1	340.9	46.364
Total			100.00	304.5	735.4	100.000

Chiral prepared using catalyst **3**

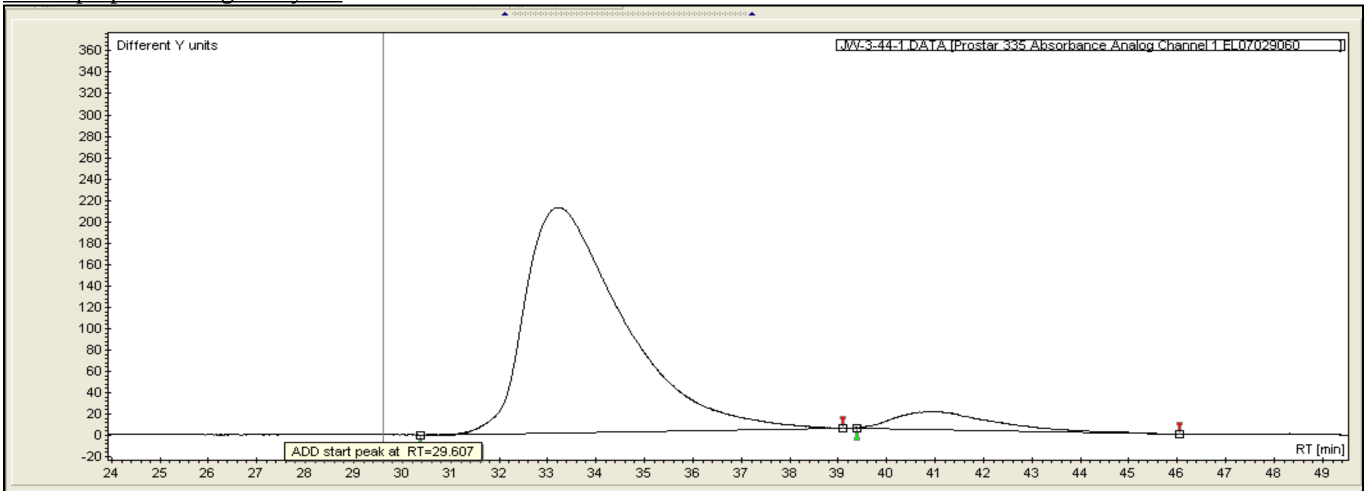

#	Name	Time [Min]	Quantity [% Area]	Height [mAU]	Area [mAU.Min]	Area % [%]
1	UNKNOWN	33.20	93.00	210.3	500.1	92.997
2	UNKNOWN	40.92	7.00	16.7	37.7	7.003
Total			100.00	227.0	537.7	100.000

Chiral prepared using catalyst **4a**

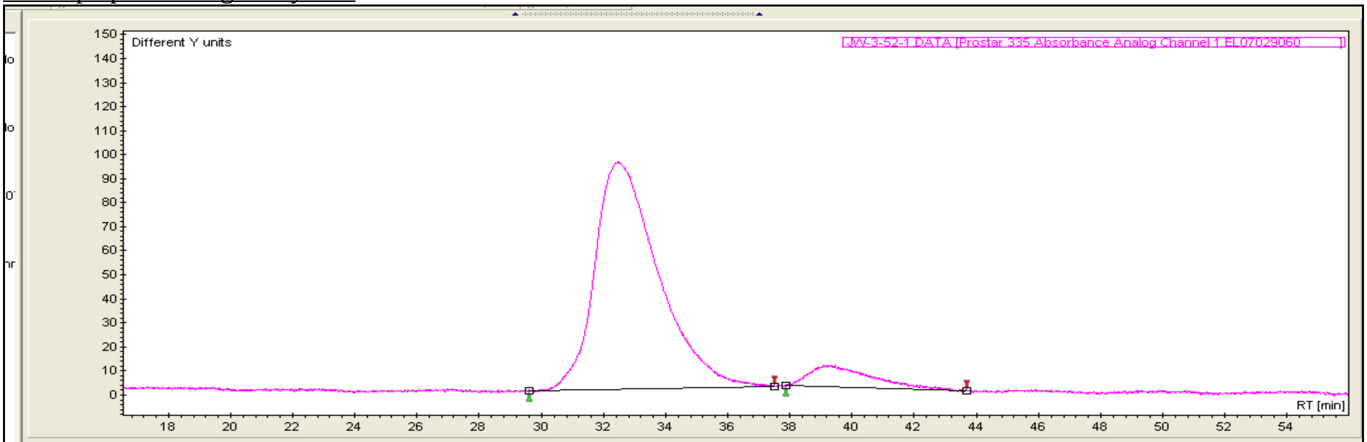

#	Name	Time [Min]	Quantity [% Area]	Height [mAU]	Area [mAU.Min]	Area % [%]
1	UNKNOWN	32.47	91.45	94.8	223.3	91.451
2	UNKNOWN	39.25	8.55	8.8	20.9	8.549
Total			100.00	103.6	244.2	100.000

(*S*)-3,3-Dimethyl-2-(nitromethyl)butanenitrile (6d) ^{1}H NMR spectrum

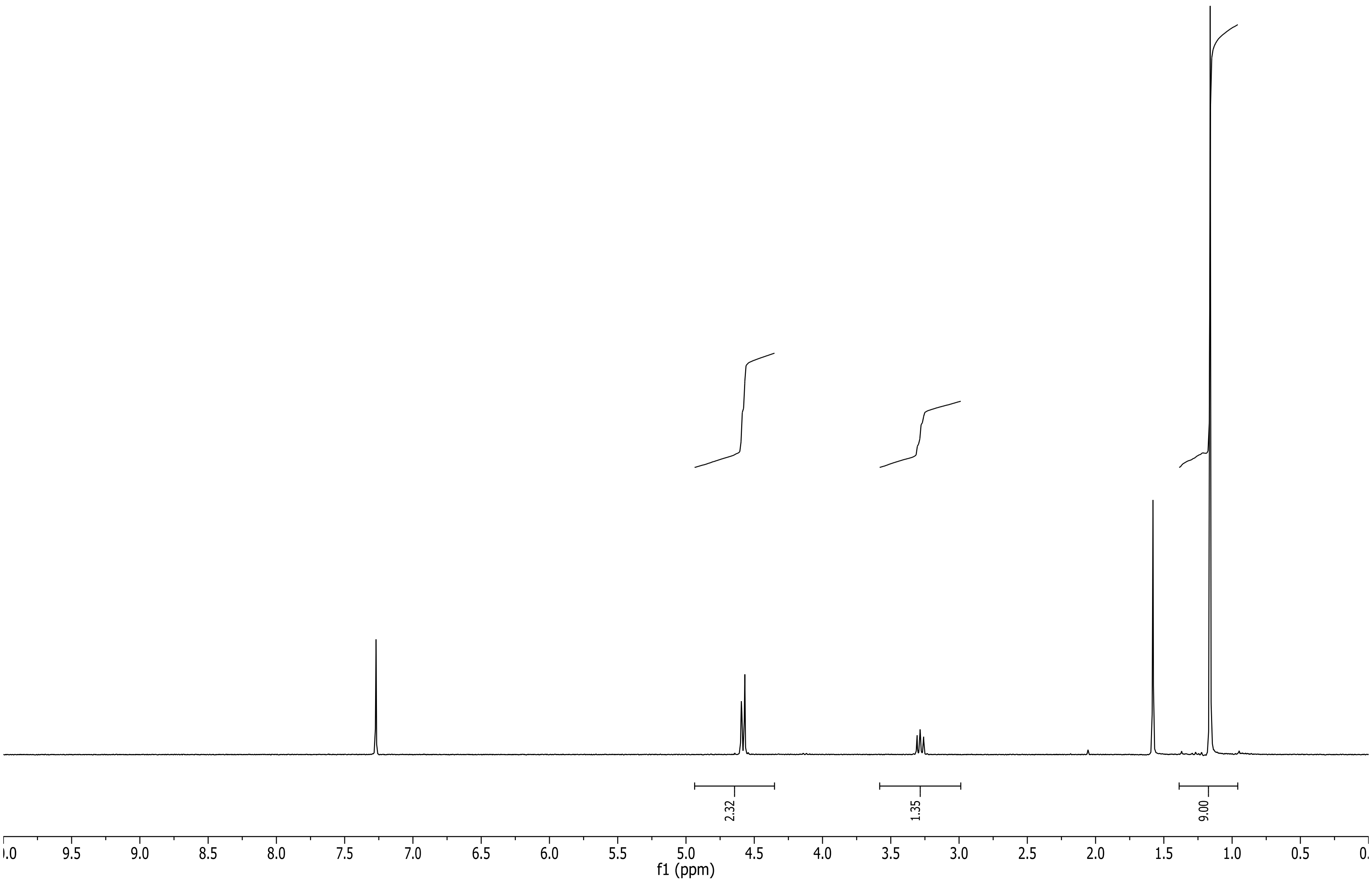

(*S*)-3,3-Dimethyl-2-(nitromethyl)butanenitrile (6d) ^{13}C NMR spectrum

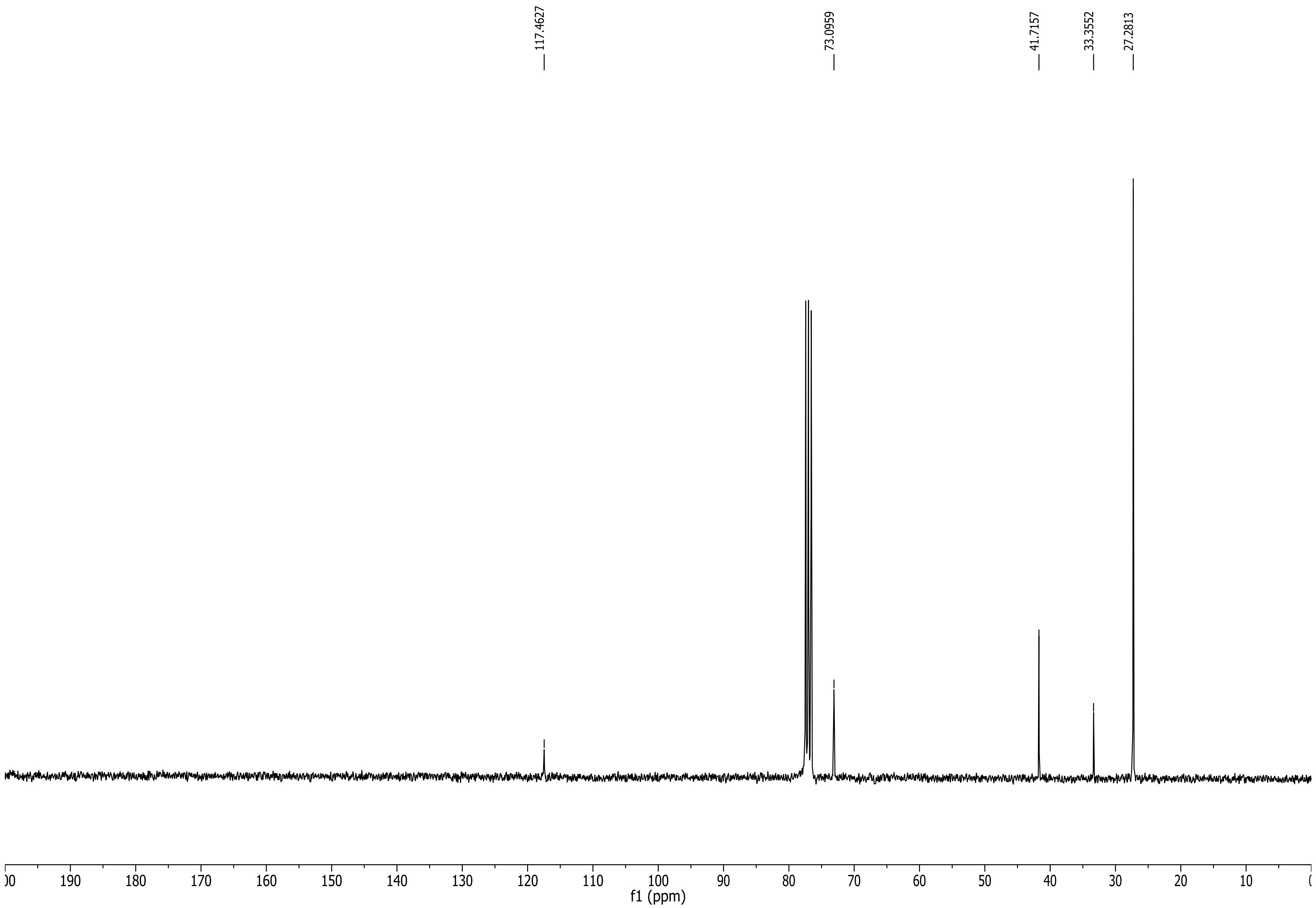

(*S*)-3,3-Dimethyl-2-(nitromethyl)butanenitrile (6d) Chiral HPLC traces

Racemic

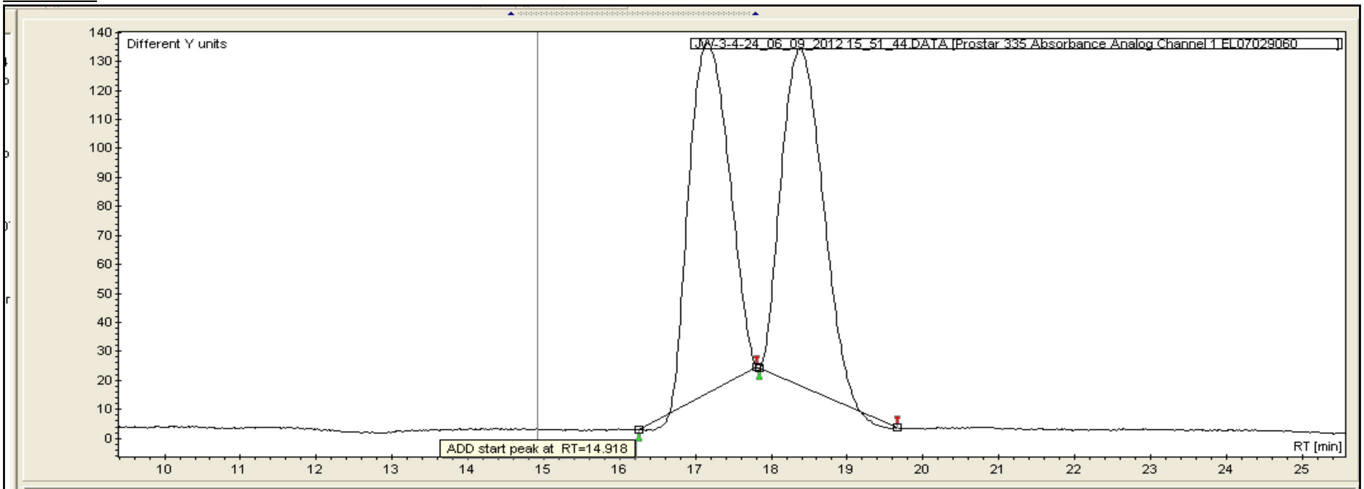

#	Name	Time [Min]	Quantity [% Area]	Height [mAU]	Area [mAU.Min]	Area % [%]
1	UNKNOWN	17.16	49.84	120.7	73.2	49.839
2	UNKNOWN	18.39	50.16	116.2	73.6	50.161
Total			100.00	236.8	146.8	100.000

Chiral prepared using catalyst **3**

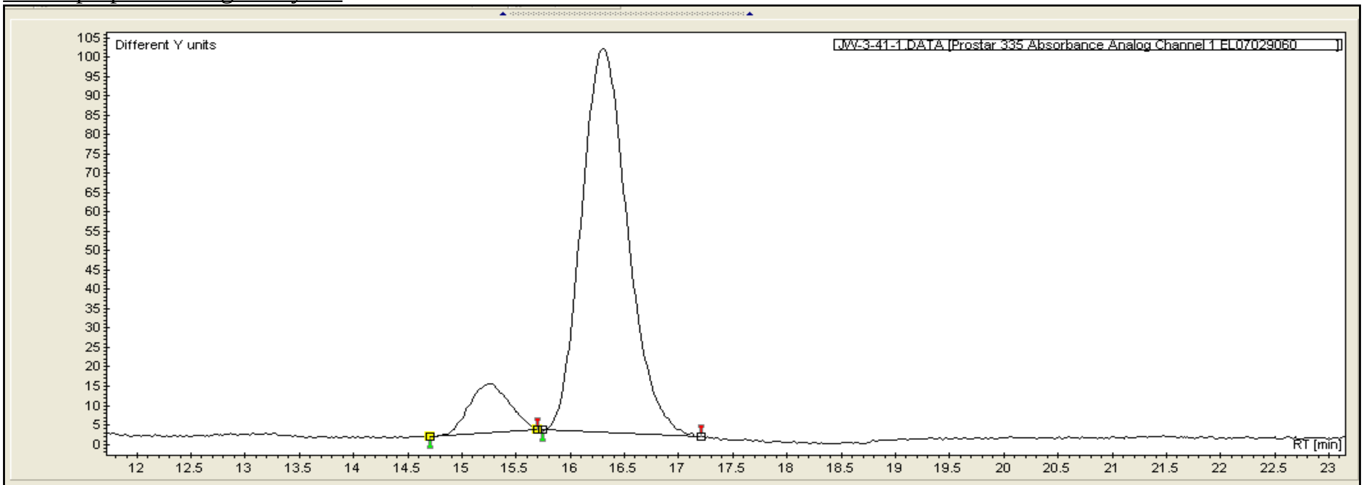

#	Name	Time [Min]	Quantity [% Area]	Height [mAU]	Area [mAU.Min]	Area % [%]
1	UNKNOWN	15.25	9.91	12.7	5.4	9.909
2	UNKNOWN	16.29	90.09	98.9	48.9	90.091
Total			100.00	111.6	54.3	100.000

Chiral prepared using catalyst **4a**

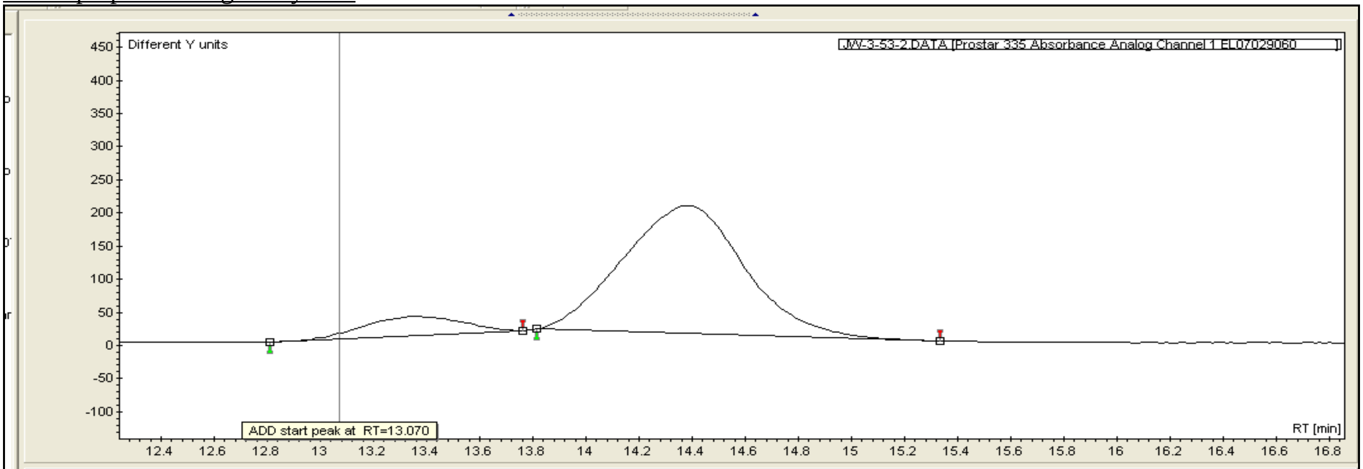

#	Name	Time [Min]	Quantity [% Area]	Height [mAU]	Area [mAU.Min]	Area % [%]
2	UNKNOWN	13.35	10.72	29.0	12.3	10.723
1	UNKNOWN	14.39	89.28	192.5	102.0	89.277
Total			100.00	221.5	114.3	100.000

(*S*)-2-(Nitromethyl)hexanonitrile (6e) ^{1}H NMR spectrum

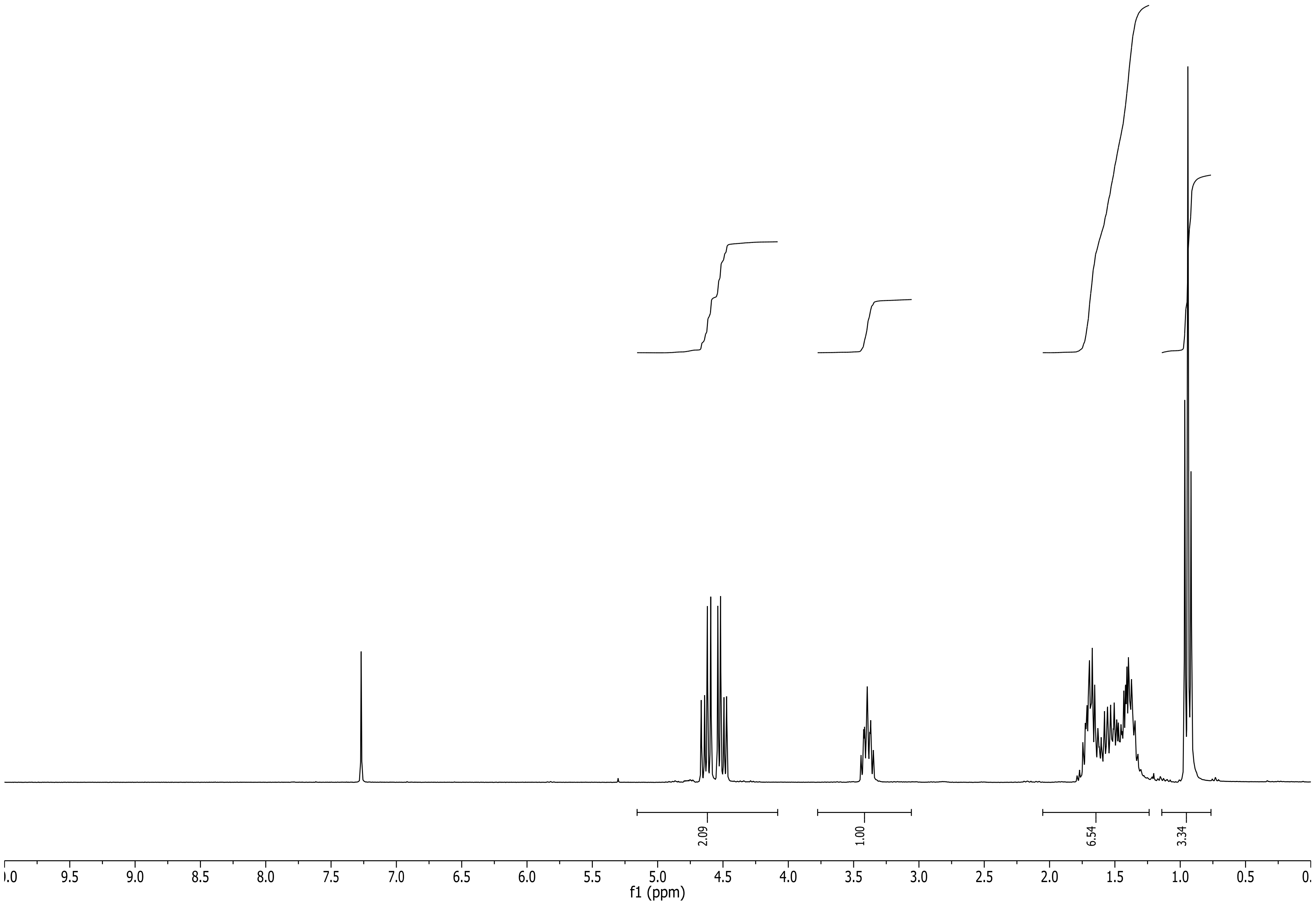

(*S*)-2-(Nitromethyl)hexanonitrile (6e) ^{13}C NMR spectrum

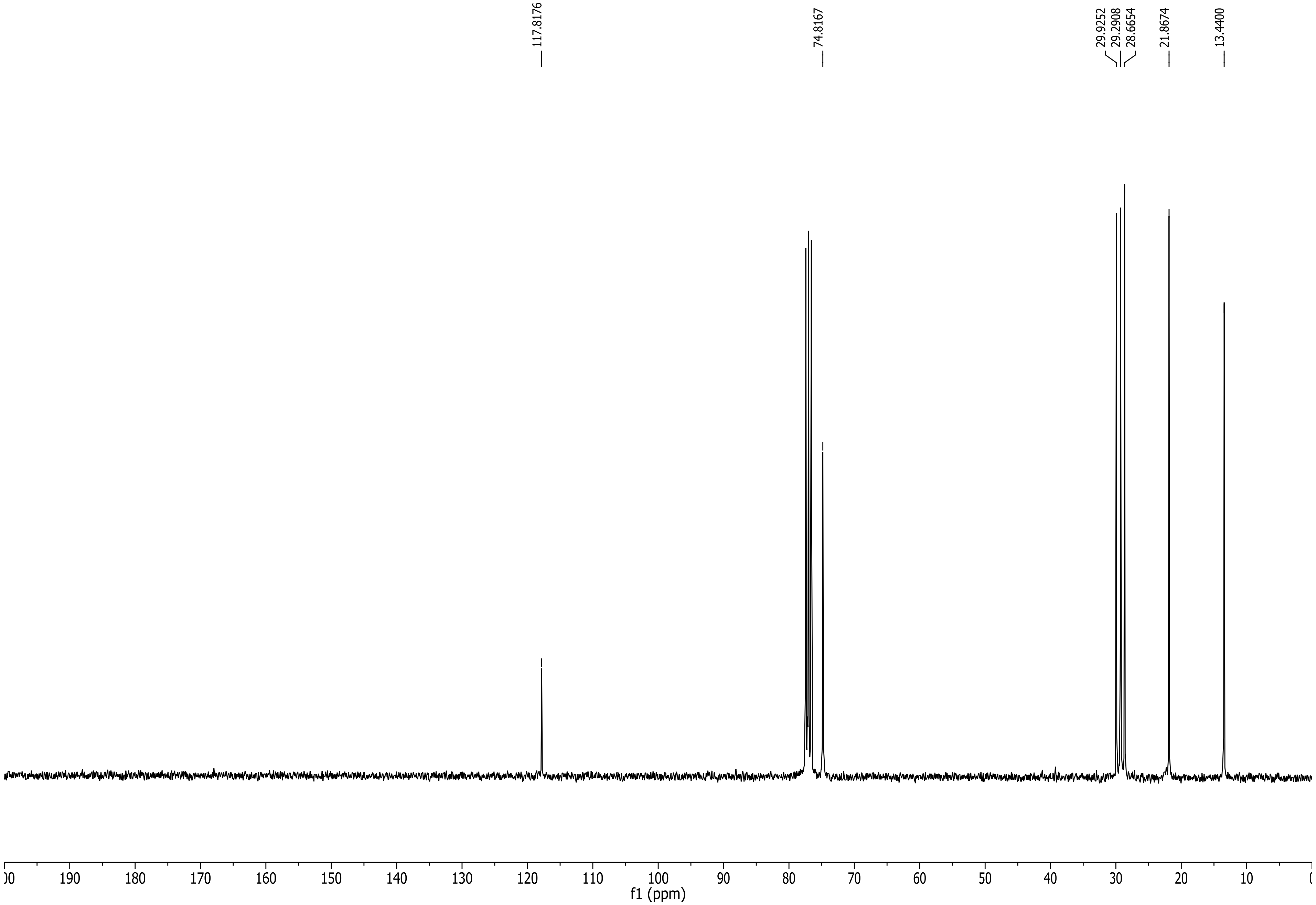

(*S*)-2-(Nitromethyl)hexanonitrile (6e) Chiral HPLC traces

Racemic

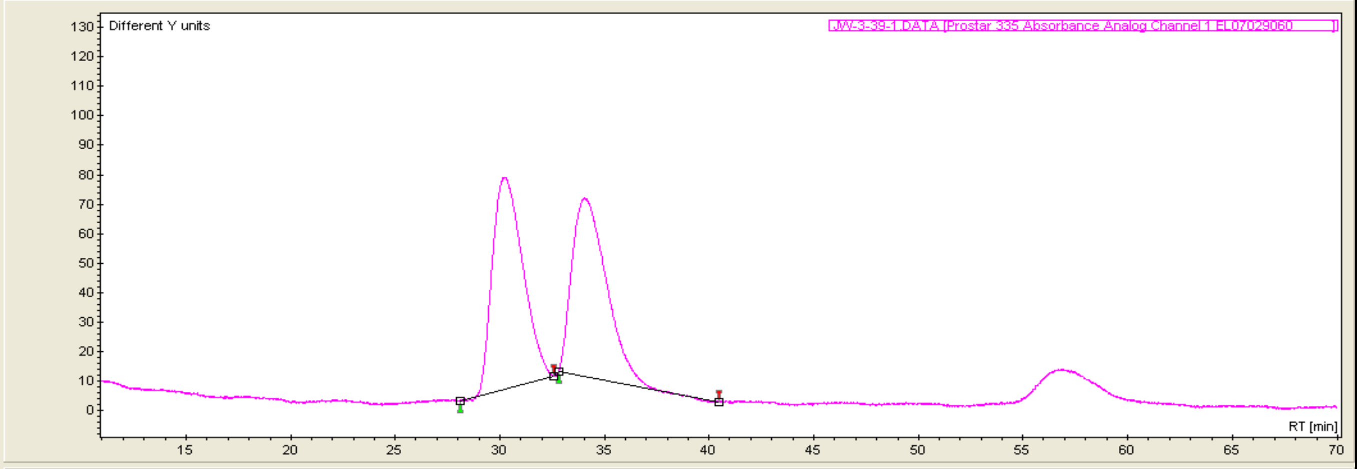

#	Name	Time [Min]	Quantity [% Area]	Height [mAU]	Area [mAU.Min]	Area % [%]
1	UNKNOWN	30.19	51.16	71.9	121.1	51.157
2	UNKNOWN	34.05	48.84	60.7	115.6	48.843
Total			100.00	132.6	236.7	100.000

Chiral prepared using catalyst **3**

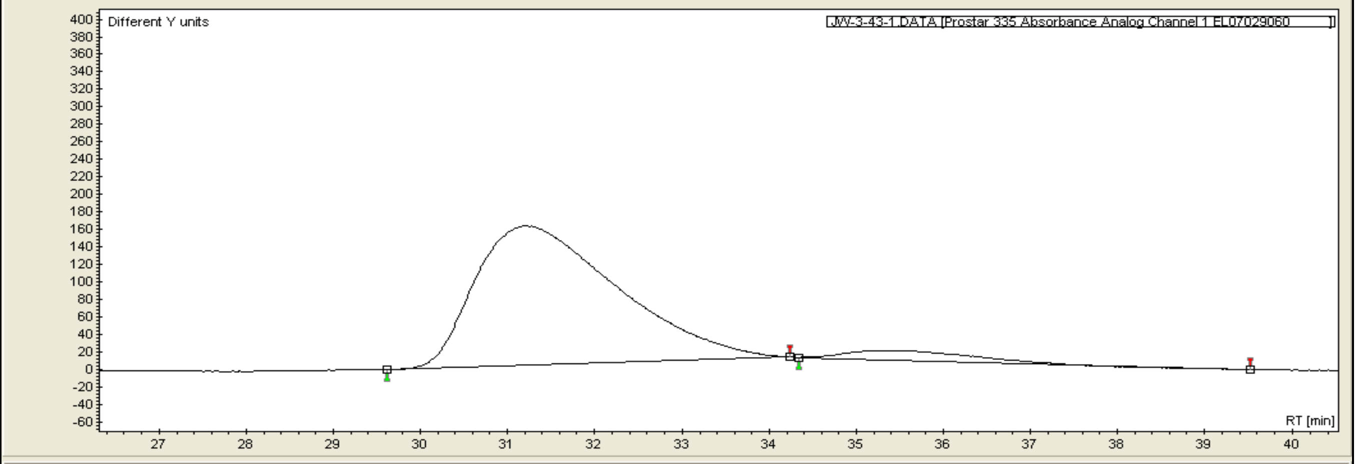

#	Name	Time [Min]	Quantity [% Area]	Height [mAU]	Area [mAU.Min]	Area % [%]
1	UNKNOWN	31.20	94.59	158.9	300.2	94.587
2	UNKNOWN	35.55	5.41	10.7	17.2	5.413
Total			100.00	169.7	317.4	100.000

Chiral prepared using catalyst **4a**

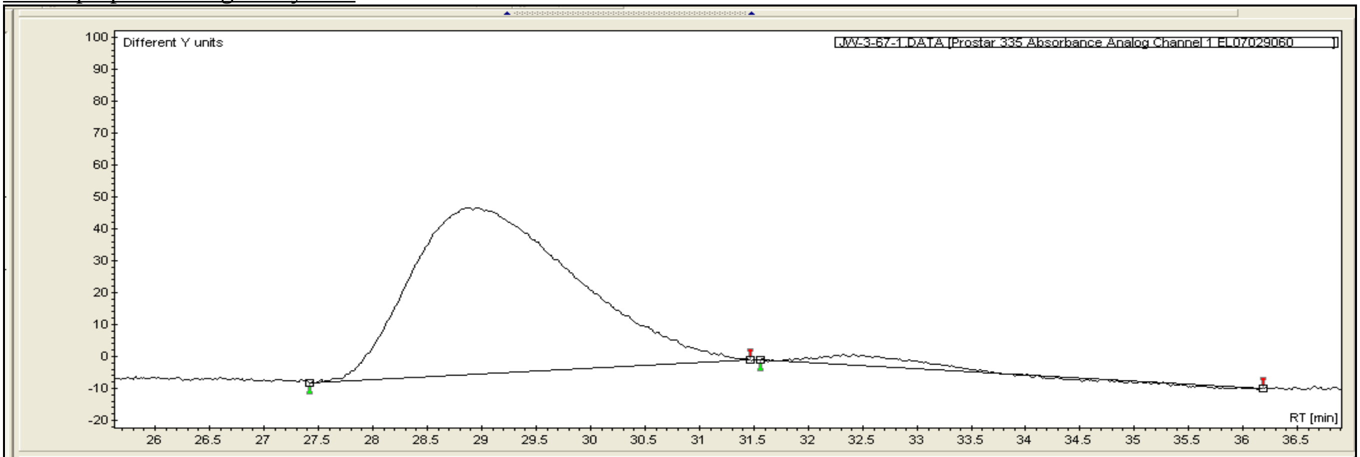

#	Name	Time [Min]	Quantity [% Area]	Height [mAU]	Area [mAU.Min]	Area % [%]
1	UNKNOWN	28.85	97.13	52.3	92.5	97.127
2	UNKNOWN	32.44	2.87	3.1	2.7	2.873
Total			100.00	55.4	95.2	100.000

(*S*)-2-(Nitromethyl)butanonitrile (6f) ^{1}H NMR spectrum

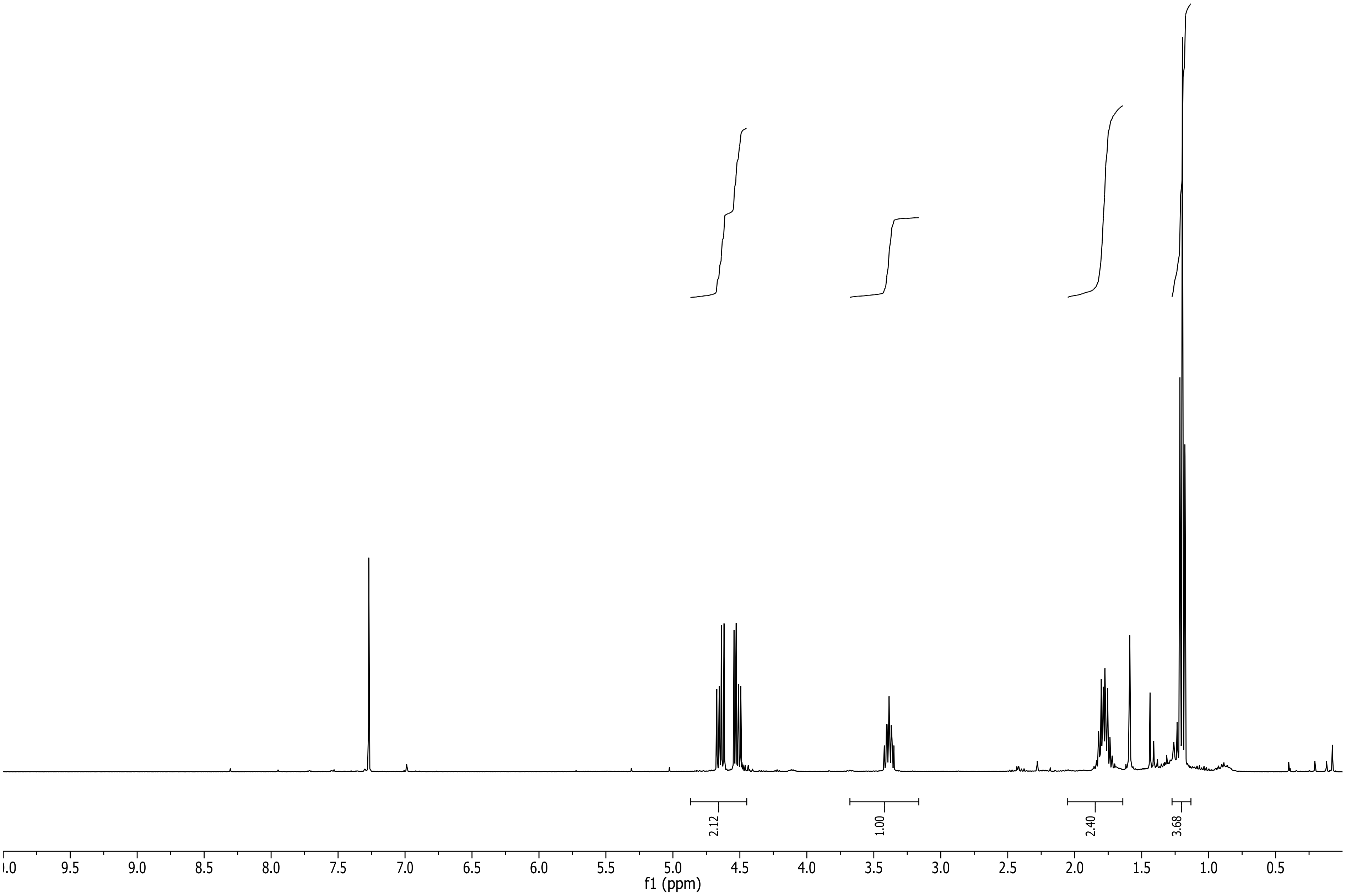

(*S*)-2-(Nitromethyl)butanonitrile (6f) ^{13}C NMR spectrum

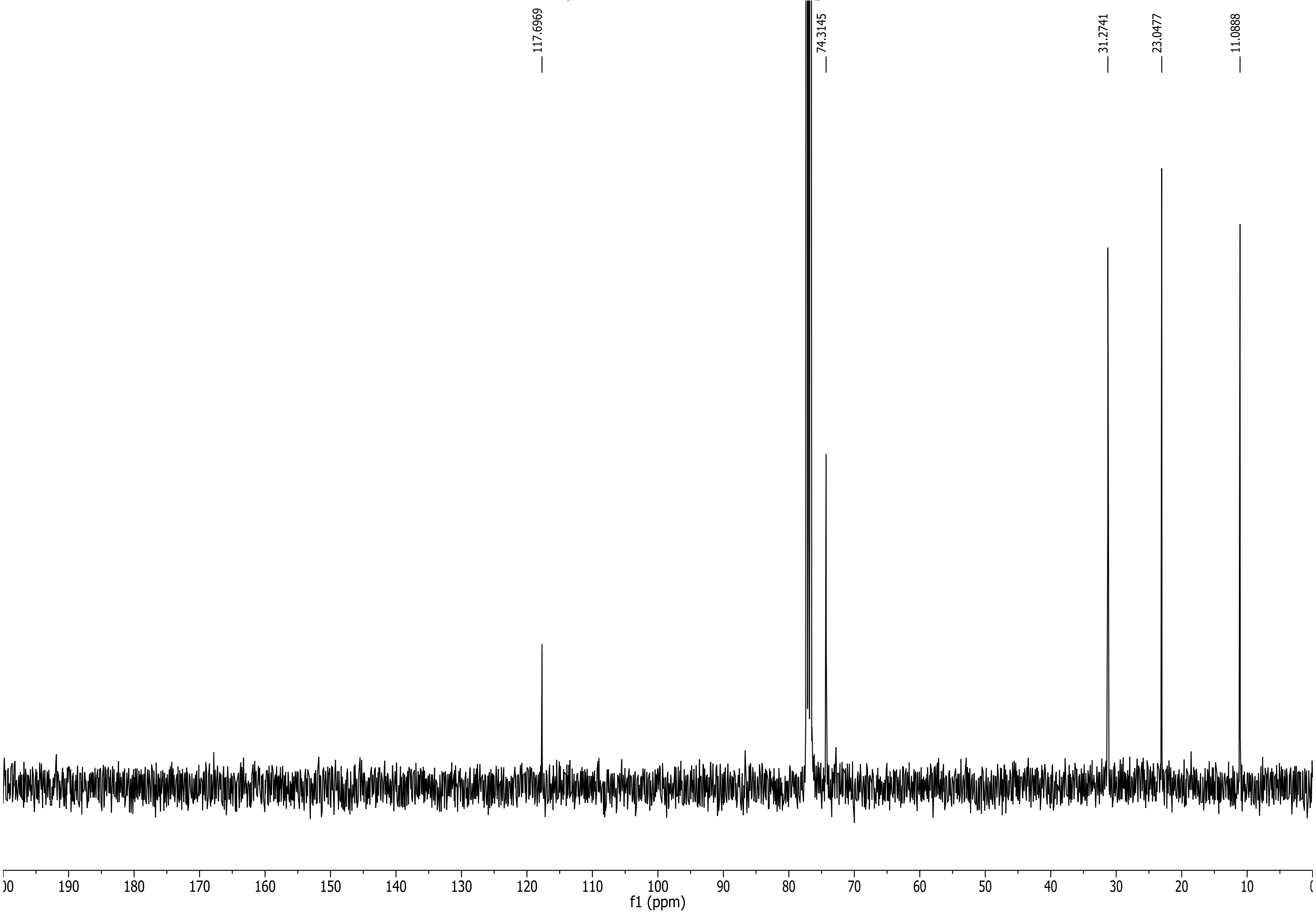

(*S*)-2-(Nitromethyl)butanonitrile (6f) Chiral HPLC traces

Racemic

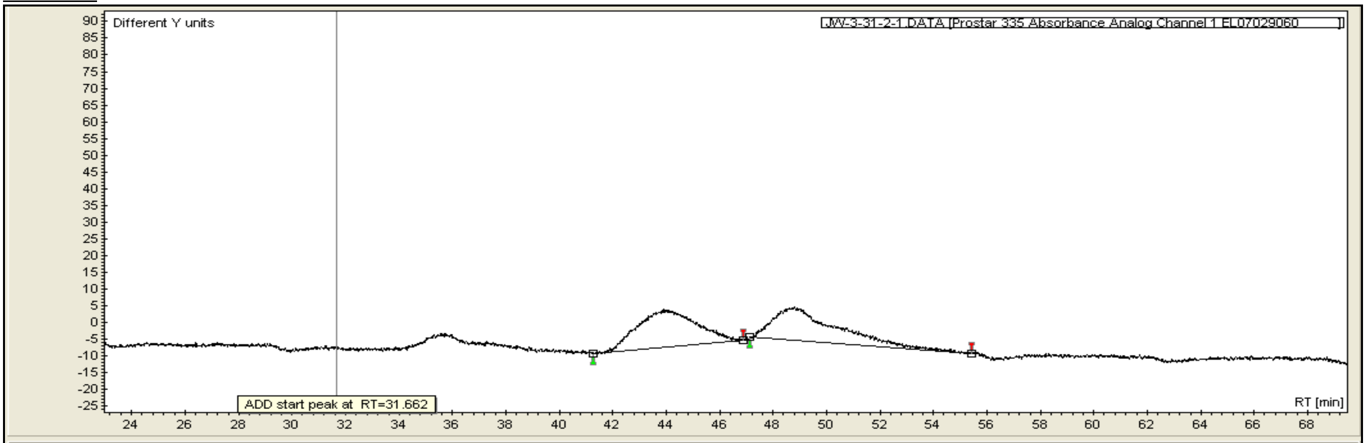

#	Name	Time [Min]	Quantity [% Area]	Height [mAU]	Area [mAU.Min]	Area % [%]
1	UNKNOWN	43.89	51.63	11.4	28.2	51.627
2	UNKNOWN	48.80	48.37	9.9	26.4	48.373
Total			100.00	21.2	54.6	100.000

Chiral prepared using catalyst **3**

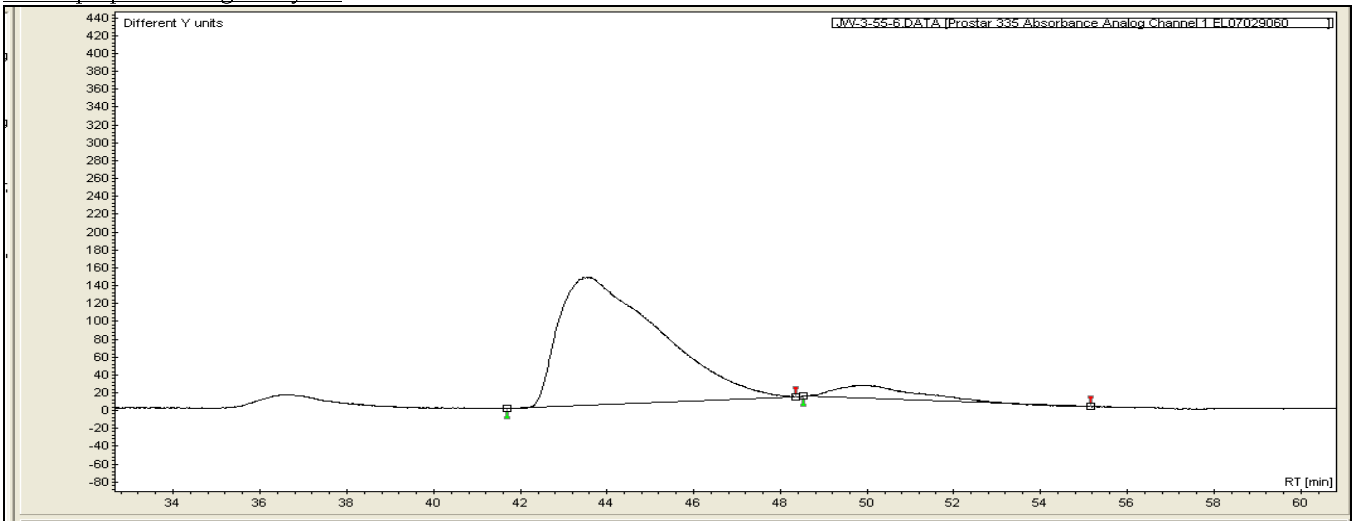

#	Name	Time [Min]	Quantity [% Area]	Height [mAU]	Area [mAU.Min]	Area % [%]
1	UNKNOWN	43.53	92.25	143.8	386.3	92.249
2	UNKNOWN	49.92	7.75	14.7	32.5	7.751
Total			100.00	158.5	418.7	100.000

Chiral prepared using catalyst **4a**

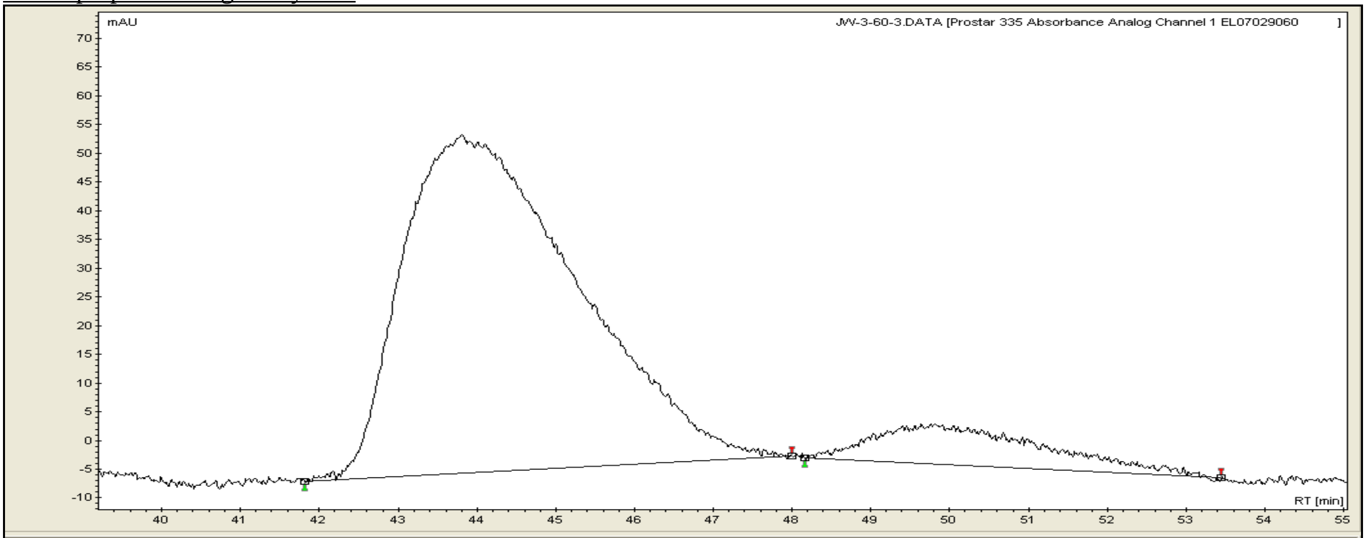

#	Name	Time [Min]	Quantity [% Area]	Height [mAU]	Area [mAU.Min]	Area % [%]
1	UNKNOWN	43.80	89.46	58.9	151.2	89.465
2	UNKNOWN	49.84	10.54	6.8	17.8	10.535
Total			100.00	65.7	169.0	100.000
